# R-spondin/YAP axis promotes gastric oxyntic gland regeneration and *Helicobacter pylori*–associated metaplasia in mice

**DOI:** 10.1172/JCI151363

**Published:** 2022-11-01

**Authors:** Anne-Sophie Fischer, Stefanie Müllerke, Alexander Arnold, Julian Heuberger, Hilmar Berger, Manqiang Lin, Hans-Joachim Mollenkopf, Jonas Wizenty, David Horst, Frank Tacke, Michael Sigal

**Affiliations:** 1 Department of Hepatology and Gastroenterology, Charité-Universitätsmedizin Berlin, Berlin, Germany.; 2 Berlin Institute for Medical Systems Biology, Max Delbrück Center for Molecular Medicine, Berlin, Germany.; 3 Berlin Institute of Health, Berlin, Germany.; 4 Department of Pathology, Charité-Universitätsmedizin Berlin, Berlin, Germany.; 5 Max Planck Institute for Infection Biology, Berlin, Germany.

**Keywords:** Gastroenterology, Stem cells, Adult stem cells, Bacterial infections, Gastric cancer

## Abstract

The stomach corpus epithelium is organized into anatomical units that consist of glands and pits. Mechanisms that control the cellular organization of corpus glands and enable their recovery upon injury are not well understood. R-spondin 3 (RSPO3) is a WNT-signaling enhancer that regulates stem cell behavior in different organs. Here, we investigated the function of RSPO3 in the corpus during homeostasis, upon chief and/or parietal cell loss, and during chronic *Helicobacter pylori* infection. Using organoid culture and conditional mouse models, we demonstrate that RSPO3 is a critical driver of secretory cell differentiation in the corpus gland toward parietal and chief cells, while its absence promoted pit cell differentiation. Acute loss of chief and parietal cells induced by high dose tamoxifen — or merely the depletion of LGR5^+^ chief cells — caused an upregulation of RSPO3 expression, which was required for the initiation of a coordinated regenerative response via the activation of yes-associated protein (YAP) signaling. This response enabled a rapid recovery of the injured secretory gland cells. However, in the context of chronic *H. pylori* infection, the R-spondin–driven regeneration was maintained long term, promoting severe glandular hyperproliferation and the development of premalignant metaplasia.

## Graphical abstract

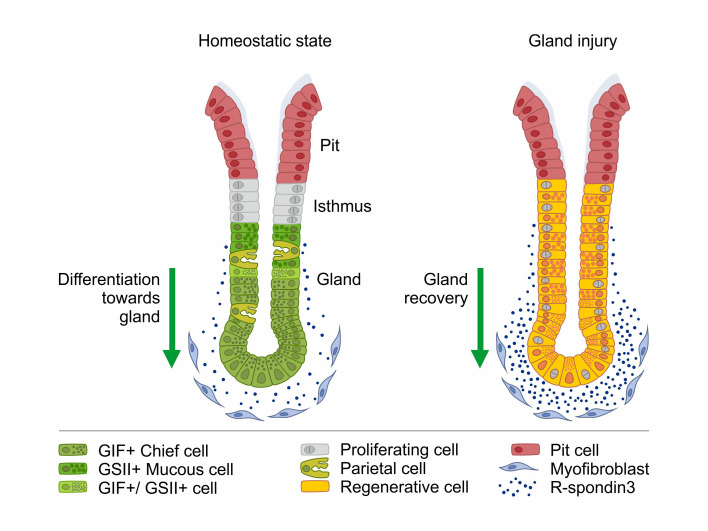

## Introduction

The epithelium of the stomach corpus is organized into clonal units that are surrounded by myosin heavy chain 11–expressing (MYH11^+^) stromal cells. These units contain multiple differentiated cell types that are compartmentalized along the base lumen axis. Under homeostatic conditions, the epithelium undergoes a constant turnover driven by highly proliferative isthmus stem cells (1, 2) that give rise to the gland cells, such as mucous neck cells, parietal cells, and chief cells as well as surface mucous pit cells (3, 4). Zymogenic chief cells are located in the base of corpus glands and secrete digestive proenzymes, such as pepsinogen C (PGC) and gastric intrinsic factor (GIF) (5).

Acute and chronic perturbations of corpus gland homeostasis are associated with characteristic alterations of the gland architecture, such as a loss of fully differentiated chief and parietal cells and an accumulation of cells coexpressing both chief cell and mucous cell markers. In the context of chronic *Helicobacter pylori* infection, such changes are linked to increased cancer risk and are termed spasmolytic polypeptide–expressing metaplasia (SPEM) (6–9).

Signals that control epithelial differentiation in the gland under homeostatic conditions and their responses to injury are not well understood. We have previously shown that in the stomach antrum and the colon, MYH11^+^ myofibroblast–derived R-spondin 3 (RSPO3), a WNT agonist that binds to LGR5 and prevents ubiquitination of Wnt receptors, controls LGR5 expression and drives LGR5^+^ stem cell turnover (10–12). Since cells in the gland base of the corpus also express LGR5, we asked how RSPO3 affects gland composition, injury repair, and epithelial responses to *H*. *pylori* in the corpus.

## Results

### RSPO3 controls epithelial differentiation toward the gland base in the corpus.

**Figure 1:**
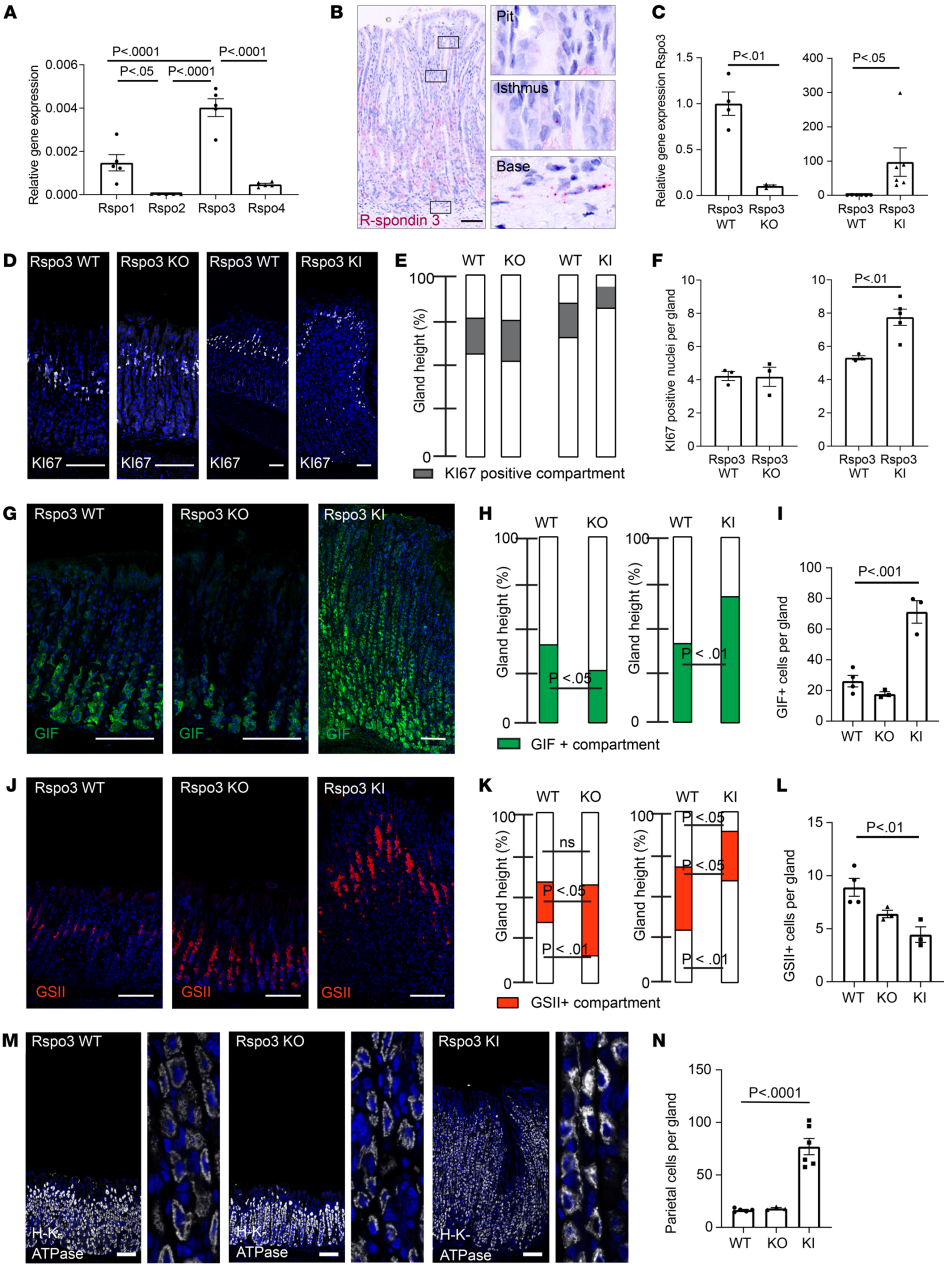
RSPO3 controls secretory cell differentiation in corpus glands. (**A**) qPCR for expression of *Rspo1–4* in Bl6 mice (*n* = 4). (**B**) ISH of *Rspo3* (red) in corpus tissue of a nontreated *MYH11-CreERT2; Rspo3^WT/WT^* control mouse. (**C**) qPCR for *Rspo3* expression in *Rspo3*-KO mice (*n* = 3) versus littermate controls (*n* = 4) and *MYH11CreERT2-Rosa26CagRspo3* (*Rspo3* KI) (*n* = 6) versus littermate controls (*n* = 6). (**D**) Immunofluorescence labeling for Ki67 (white) representative of *Rspo3*-KO and *Rspo3*-KI mice and littermate controls. (**E**) Location and relative size of the Ki67-positive gland compartment. (**F**) Quantification of the number of Ki67^+^ nuclei per gland in *Rspo3*-KO and *Rspo3*-KI mice and corresponding littermate controls (*n* = 3 mice per group). (**G**) Immunofluorescence labeling for GIF (green) representative of *Rspo3*-KO and *Rspo3*-KI mice and littermate controls. (**H**) Location and relative size of GIF-positive gland compartment. (**I**) Quantification of the number of GIF^+^ cells per gland in *Rspo3*-KO and *Rspo3*-KI mice and corresponding littermate controls (*n* = 3 mice per group). (**J**) Immunofluorescence labeling for GSII (red) representative of *Rspo3*-KO and *Rspo3*-KI mice and littermate controls. (**K**) Location and relative size of the GSII-positive gland compartment. (**L**) Quantification of the number of GSII^+^ cells per gland in *Rspo3*-KO and *Rspo3*-KI mice and corresponding littermate controls (*n* = 3 mice per group). (**M**) Immunofluorescence images of H/K-ATPase labeling (gray) in sections representative of *Rspo3*-KO and *Rspo3-*KI mice and corresponding littermate controls. (**N**) Quantification of parietal cells per gland in *Rspo3*-KO and *Rspo3*-KI mice and corresponding littermate controls (*n* = 3 mice per group). Mice were treated with tamoxifen 2 weeks before euthanasia. Scale bars: 100 μm. Enlargements in **M** equal 8:1 magnification. Unpaired parametric *t* test (**C**, **E**, **F**, **H**, and **K**); 1-way ANOVA with Tukey’s multiple-comparison test (**A**, **I**, and **L**).

RSPO3 is an enhancer of *WNT* signaling and a critical regulator of intestinal and antral LGR5^+^ stem cell homeostasis (11–15), but its role in the stomach corpus glands has not been investigated in detail. To this end, we first applied quantitative PCR (qPCR) analysis and in situ hybridization to examine the expression pattern of the R-spondin family in the corpus. As in the antrum, RSPO3 was the most abundant homolog in the corpus (Figure 1A), and its expression was most prominent in the stroma beneath the gland base (Figure 1B), where MYH11^+^ myofibroblasts reside. No *Rspo3* expression was found adjacent to the gland pit and only rare expression adjacent to the gland isthmus (Figure 1B).

To address the physiological function of RSPO3 in the corpus, we generated mice to conditionally deplete *Rspo3* expression specifically in MYH11^+^ myofibroblasts (*MYH11-CreERT2; Rspo3^fl/fl^*, hereafter referred to as *Rspo3* KO) or conditionally induce overexpression of *Rspo3* (*MYH11-CreERT2/Rosa26Sor6[CAG–Rspo3]*, hereafter referred to as *Rspo3* knockin [KI]) (Supplemental Figure 1, A and B; supplemental material available online with this article; https://doi.org/10.1172/JCI151363DS1). qPCR of corpus tissue 2 months after depletion of *Rspo3* in MYH11^+^ cells revealed that *Rspo3* expression was reduced by 90%, confirming that MYH11^+^ cells are the main source of *Rspo3* in the corpus (Figure 1C). In addition, qPCR revealed that downregulation of *Rspo3* expression did not result in compensatory upregulation of other R-spondin isoforms in the corpus (Supplemental Figure 1, C–E). *Rspo3* KI induced in MYH11^+^ cells led to significantly increased *Rspo3* expression (Figure 1C). Of note, despite a 90-fold increase of expression in *Rspo3*-overexpressing mice, *Rspo3* expression was still concentrated in the cells beneath the gland base (Supplemental Figure 1F), and expression of the RSPO3 target gene *LGR5* was similarly concentrated in the gland base (Supplemental Figure 1G).

To assess changes in gland architecture upon *Rspo3* depletion or overexpression, we performed H&E staining of corpus sections and found that, despite altered RSPO3 levels, the overall architecture of the glands was maintained (Supplemental Figure 2A). However, the height of the glands was altered, with depletion of *Rspo3* reducing and overexpression increasing gland height (Supplemental Figure 2B).

To determine whether the differences in gland height were primarily driven by altered proliferative activity, we labeled the corpus tissue for the proliferation marker Ki67 (Figure 1D). The proliferative compartment of WT mice was located in the isthmus region of the glands. In mice lacking *Rspo3*, the location and the height of the Ki67^+^ compartment as well as the number of Ki67^+^ cells within this compartment resembled that in glands of WT mice (Figure 1, E and F). In *Rspo3*-KI mice, the Ki67^+^ compartment was also located in the isthmus of the gland, but the number of Ki67^+^ cells was significantly increased compared with that in controls (Figure 1F). However, the proportion of the gland harboring Ki67^+^ cells was smaller (Figure 1E). We concluded that the gland enlargement upon *Rspo3* overexpression resulted from an enrichment of nonproliferating cells. We therefore analyzed the abundance of differentiated cell types. Immunofluorescence labeling and quantitative analysis revealed that the MUC5AC^+^ pit compartment was reduced in *Rspo3-*KI mice and increased in *Rspo3*-KO mice compared with controls (Supplemental Figure 2, C and D).

Next, we evaluated the expression pattern of 2 classic chief cell markers: PGC (Supplemental Figure 2, E and F) and GIF (Figure 1, G–I). Both the proportion of the GIF^+^ cell compartment relative to the rest of the gland (Figure 1H) and the absolute number of GIF^+^ cells per gland (Figure 1I) were increased upon overexpression of *Rspo3* and diminished upon *Rspo3* loss. Although the PGC^+^ compartment was not significantly altered in *Rspo3*-KO mice compared with controls, it was increased in *Rspo3*-KI mice, further confirming an increase of chief cells upon overexpression of *Rspo3* (Supplemental Figure 2, E and F). In addition, immunofluorescence staining revealed a higher number of Mist1^+^ base cells upon *Rspo3* overexpression, confirming that RSPO3 not only increases the number of chief cells, but also drives their terminal differentiation (Supplemental Figure 2, G and H).

RSPO3 has been shown to control *LGR5* expression in the gastrointestinal tract (11, 12, 15), and LGR5 is a marker of gland base chief cells (16, 17). qPCR in corpus revealed that *LGR5* was significantly downregulated upon *Rspo3* depletion and upregulated in *Rspo3*-KI mice (Supplemental Figure 2I). Transcriptome analysis from the corpus of *Rspo3*-KI mice and subsequent Gene Set Enrichment Analysis (GSEA) revealed that *Rspo3* overexpression indeed induced expression of various stem cell–associated genes, resulting in a significant positive enrichment for “*LGR5* stem cell signature” (18) genes (Supplemental Figure 2J).

In contrast with chief cell markers, the abundance of the GSII^+^ mucous neck compartment was increased in *Rspo3*-KO mice and reduced in *Rspo3*-KI mice (Figure 1, J–L). Furthermore, we noticed that the GSII^+^ compartment was shifted toward the gland surface upon *Rspo3* overexpression and toward the gland base upon *Rspo3* depletion (Figure 1K). Additional quantitative analysis revealed that the number of GIF^+^GSII^+^ cells, which represent cells in the process of differentiation from GSII^+^ mucous neck cells to GIF^+^ chief cells (4), was significantly increased in *Rspo3*-overexpressing mice (Supplemental Figure 2K). Parietal cells were found throughout the gland in WT, *Rspo3*-KO, and *Rspo3*-KI mice (Figure 1, M and N) with no obvious changes in distribution, but an absolute increase of parietal cell numbers in *Rspo3*-overexpressing mice. Together, these data indicate that RSPO3 affects the differentiation dynamics of the corpus epithelium by promoting differentiation into glandular lineages, including chief cells and parietal cells, which occurs at the expense of pit cell differentiation.

### RSPO3 induces glandular cell differentiation in organoids.

**Figure 2:**
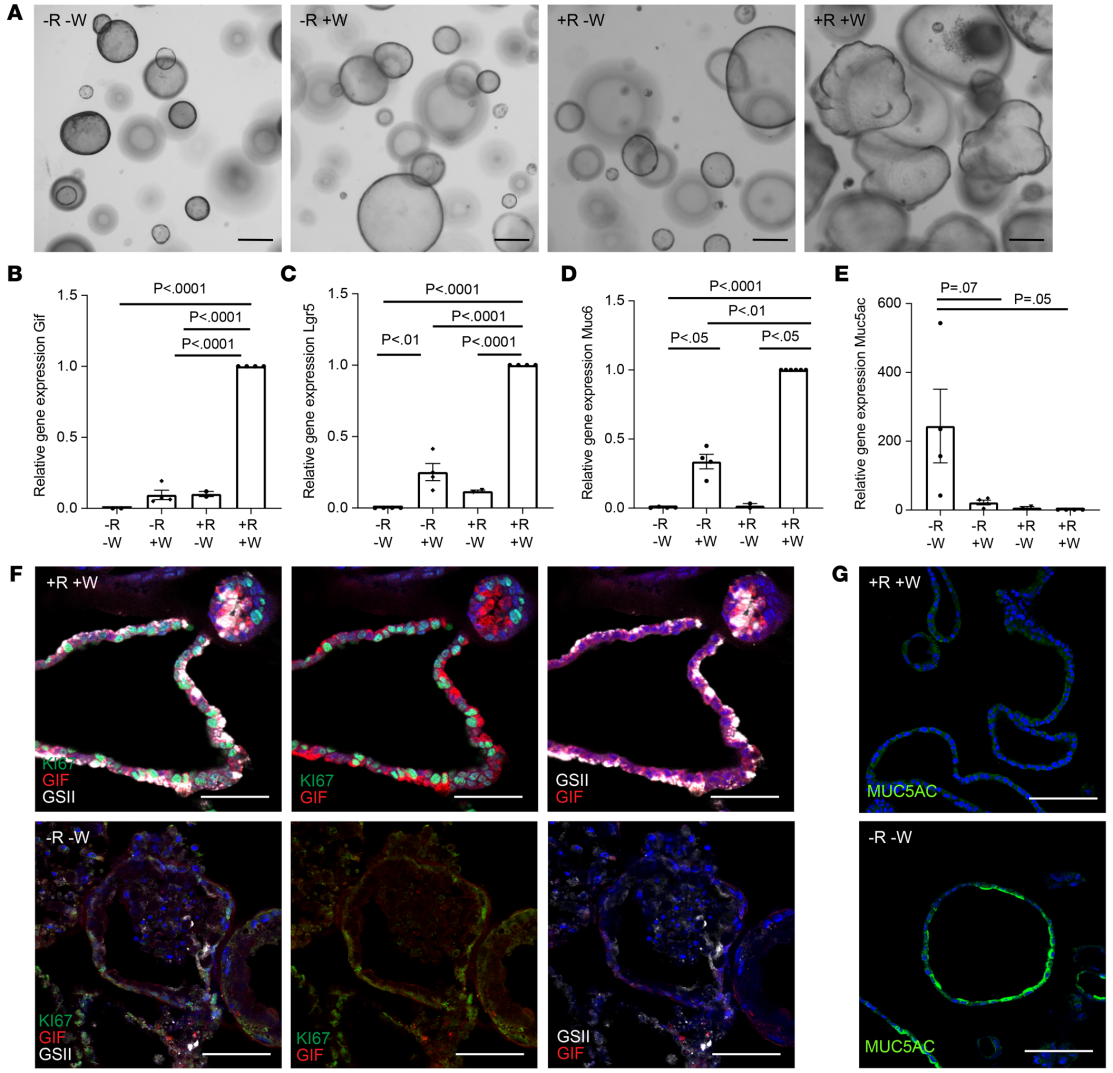
RSPO3 induces glandular cell differentiation in organoids. All organoids were grown from the murine corpus and kept in 10% RSPO1-conditioned medium. (**A**) Representative images of organoids grown in –R–W, –R+W, +R–W, and +R+W conditioned media. Scale bars: 500 μm. (**B**–**E**) qPCR data showing expression of (**B**) *GIF,* (**C**) *LGR5*, (**D**) *Muc6*, and (**E**) *Muc5ac* in organoids grown in –R–W (4 replicates from 2 mice), –R+W (4 replicates from 2 mice), +R–W (2 replicates from 2 mice), and +R+W media (4 replicates from 2 mice). (**F**) Representative images of sections from organoids grown in +R+W and –R–W media stained for Ki67 (green), GIF (red), and GSII (white). (**G**) Representative images of sections from organoids grown in +R+W and –R–W media stained for MUC5AC (green). Scale bars: 50 μm. One-way ANOVA with Tukey’s multiple-comparison test.

To explore whether R-spondin–driven effects on epithelial differentiation are directly mediated via its interaction with the epithelium, we used 3D organoids from murine primary corpus cells. Organoid growth relies on the addition of exogenous growth factors, including RSPO3 and WNT. To address the role of R-spondin, we established organoid cultures in full medium for 2 days, followed by continued culture in full medium (+R+W) or removal of either R-spondin (–R+W), WNT (+R–W), or both (–R–W). Morphological analysis revealed that organoids grown in +R+W medium grew larger in diameter than those grown in –R–W, –R+W, or +R–W medium and formed buds, whereas those without WNT and/or R-spondin maintained a spherical shape (Figure 2A).

We performed qPCR analysis for expression of *LGR5* as well as for the chief cell maker *GIF*, the mucous neck cell marker *Muc6,* and the surface cell maker *Muc5ac*. In +R+W conditions, expression of the glandular cell markers *LGR5*, *GIF*, and *Muc6* was significantly higher compared with in all other conditions, while in –R–W conditions, *Muc5AC* was significantly upregulated (Figure 2, B–E), indicating that WNT/R-spondin signaling induces differentiation toward the glandular lineages in organoids, while its absence causes pit cell differentiation. Organoids grown in –R+W and -W+R medium also expressed reduced levels of *LGR5*, *GIF,* and *Muc6* compared with organoids grown in +R+W medium; thus, both factors are required to maintain *LGR5* expression and to control glandular cell differentiation. Of note, an RSPO1-conditioned medium is routinely used for organoid cultures. To investigate whether RSPO1 and RSPO3 have similar effects on organoids, as has been reported in the intestine (19), we grew organoids in the standard medium as well as in medium supplemented with recombinant RSPO3 protein and compared their effect on organoid differentiation. Indeed, the morphology (Supplemental Figure 3A) as well as the organoid-forming efficiency (Supplemental Figure 3B) were similar in both conditions. In addition, we performed qPCR analysis from organoids grown in different concentrations of recombinant RSPO3 and found that increasing concentrations result in increased levels of *GIF*, *LGR5,* and *Muc6* expression, but reduced levels of *Muc5ac* expression (Supplemental Figure 3C), replicating the findings with organoids grown in RSPO1-conditioned medium. Thus, all subsequent organoid experiments were carried out using our standard medium containing 10% RSPO1-conditioned medium.

Immunofluorescence staining revealed that organoids grown in +R+W medium contained a higher proportion of Ki67^+^ cells compared with those grown in –R–W conditions (Figure 2F). Staining for GIF and the mucous neck cell marker GSII (Figure 2F) as well as for MUC5AC (Figure 2G) confirmed the findings from the qPCR analysis and demonstrated that high WNT/RSPO signaling enables differentiation into gland cells, while lack of R-spondin promotes pit cell differentiation.I t should be noted that in organoids, most GIF^+^ cells also coexpressed GSII. Furthermore, parietal cells were absent. Thus, organoids from the corpus appear to not recapitulate the fully mature corpus gland, but likely represent a more regenerative state.

### RSPO3 is upregulated upon high-dose tamoxifen–driven gland injury and promotes glandular regeneration.

**Figure 3:**
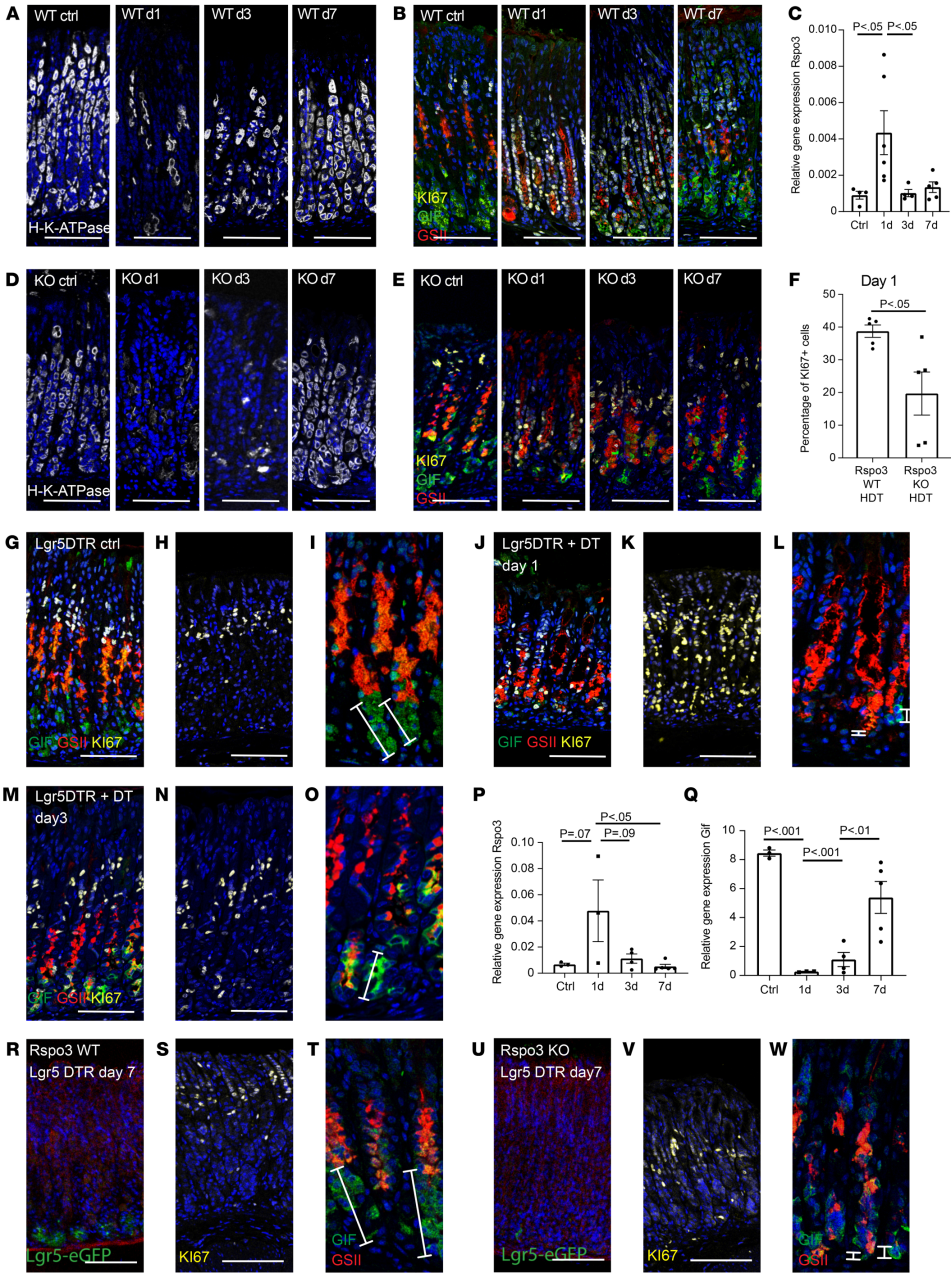
RSPO3 is upregulated upon HDT-driven gland injury and upon depletion of LGR5^+^ chief cells and promotes glandular regeneration. (**A**, **B**, **D**, **E**) Representative images of sections from (**A** and **B**) *Rspo3*-WT and (**D** and **E**) *Rspo3*-KO mice treated with HDT and sacrificed 1, 3, or 7 days later and nontreated controls stained for (**A** and **D**) H/K-ATPase (white) and (**B** and **E**) GIF (green), GSII (red), and Ki67 (yellow). (**C**) qPCR for *Rspo3* expression in *Rspo3*-WT mice treated with HDT and sacrificed on day 1 (*n* = 6), 3 (*n* = 4), or 7 (*n* = 4) versus controls (*n* = 4). (**F**) Percentage of Ki67^+^ cells/gland in *Rspo3*-WT (*n* = 5) versus *Rspo3-*KO mice (*n* = 5) on day 1 after HDT-induced injury. (**G**–**L**) LGR5DTR mice were treated with (**G**–**I**) PBS or (**J**–**L**) DT for 3 days and sacrificed 24 hours after treatment. Immunofluorescence labeling for (**G** and **J**) GIF (green), GSII (red), Ki67 (yellow), (**H** and **K**) Ki67, and (**I** and **L**) GIF and GSII on sections from PBS-treated mice. (**M**–**O**) LGR5DTR mice were treated with DT for 3 days and sacrificed 3 days after treatment. Immunofluorescence labeling for (**M**) GIF (green), GSII (red), Ki67 (yellow), (**N**) Ki67, and (**O**) GIF and GSII. (**P** and **Q**) qPCR for (**P**) *Rspo3* or (**Q**) *GIF* expression of DT-treated LGR5DTR mice sacrificed on day 1 (*n* = 5), 3 (*n* = 4), or 7 (*n* = 4 mice) versus controls (*n* = 4). (**R**–**W**) Mice were treated with tamoxifen and DT 7 days before euthanasia. (**R** and **U**) EGFP expression of agarose sections from (**R**) *LGR5-DTR/EGFP;MYH11-CreERT2;Rspo^WT/WT^* and (**U**) *LGR5-DTR/EGFP;MYH11-CreERT2;Rspo3^fl/fl^* mice. (**S**, **T**, **V**, and **W**) Representative images of sections from (**S** and **T**) *LGR5-DTR/EGFP;MYH11-CreERT2;Rspo^WT/WT^* and (**V** and **W**) *LGR5-DTR/EGFP;MYH11-CreERT2;Rspo3^fl/ft^* mice stained for (**S** and **V**) Ki67 (yellow) and (**T** and **W**) GIF (green) and GSII (red). Scale bars: 100 μm. Enlargements in **I**, **L**, **O**, **T**, and **W** equal 2:1 magnification. Unpaired parametric *t* test (**F**), 1-way ANOVA with Tukey’s multiple-comparison test (**C**, **P**, and **Q**).

Having established RSPO3 as an important regulator of the glandular lineages during homeostasis, we next assessed its regenerative function. To this end, we applied high-dose tamoxifen (HDT) treatment, which has been demonstrated to transiently alter corpus gland homeostasis by promoting injury and almost complete depletion of parietal and chief cells (20, 21). Mice were injected intraperitoneally with 250 μg tamoxifen/g body weight on 2 consecutive days and sacrificed 1, 3, or 7 days later. As an indicator of tissue damage, we analyzed the abundance of parietal cells. We found an almost complete loss of parietal cells in the glands on day 1 after tamoxifen treatment (Figure 3A). This was accompanied by a loss of chief cell markers (Figure 3B). On day 3 there was a partial and on day 7 an almost complete restoration of parietal and chief cells (Figure 3, A and B). qPCR also revealed a loss of *LGR5* and *GIF* expression on day 1, which was almost completely restored on day 7 (Supplemental Figure 4, A and B). qPCR analysis further revealed a strong increase in expression of *Rspo3* as well as *Rspo1*, but not *Rspo2* or *Rspo4*, on day 1 (Figure 3C and Supplemental Figure 4C). This was accompanied by massive proliferation on day 1 after injury, as indicated by Ki67 staining (Figure 3B).

To evaluate the role of RSPO3 signaling for tissue responses to injury, we treated *Rspo3*-KO mice with an induction dose of tamoxifen to induce the KO, allowed them to recover, and then used HDT treatment to induce glandular cell loss using the same protocol as described above. Immunofluorescence staining revealed a loss of parietal cells similar to that observed in WT mice on day 1 (Figure 3D), but while parietal cells and the overall gland integrity were already substantially restored in WT mice on day 3, in *Rspo3*–KO mice, the parietal cells were still heavily affected on day 3 after injury. Importantly, mice that lacked *Rspo3* expression showed reduced glandular hyperproliferation upon injury compared with WT mice (Figure 3, E and F) and instead of full recovery of the chief cell compartment, gland base cells continued to coexpress GIF and GSII, indicating the lack of fully mature chief cells. Of note, the upregulation of *Rspo1* on day 1 in WT mice was also observed in *Rspo3*-KO mice compared with untreated *Rspo3*-KO mice, but did not exceed the expression level observed in WT mice (Supplemental Figure 4D). In summary, we found that R-spondin is not only necessary to maintain differentiation of glandular cells during homeostasis, but also drives their recovery upon injury. However, while chief cell maturation was fully dependent on the presence of RSPO3 signaling, parietal cells were able to recover in the absence of RSPO3 signaling, although the process was delayed compared with that in WT mice.

### Depletion of LGR5^+^ chief cells is sufficient to initiate RSPO3-driven regenerative gland responses.

Since we noticed a loss of chief cells upon tamoxifen treatment, we asked whether their loss is sufficient to promote upregulation of RSPO3 expression in the stroma. We therefore depleted LGR5^+^ cells by injecting diphtheria toxin (DT) intraperitoneally into *LGR5-DTR-eGFP* mice. Immunofluorescence revealed an almost complete loss of chief cells (labeled by GIF) in the experimental group, but not in the control group (Figure 3, G, I, J, and L). Upon treatment with DT, the remaining adjacent cells showed high proliferative activity (Figure 3, H and K). Similarly to the dynamics in the HDT model, on day 3 after injury, the proliferative activity in the tissue decreased (Figure 3, M and N) and the first signs of chief cell recovery appeared, including reexpression of the chief cell marker *GIF* (Figure 3, O and Q). qPCR for R-spondins revealed that, also in the LGR5DTR model, expression of *Rspo3* — but not the other R-spondin isoforms — was upregulated at 24 hours, indicating that loss of chief cells was sufficient to initiate *Rspo3* expression (Figure 3P and Supplemental Figure 4E).

We next generated *LGR5-DTR-eGFP; MYH11-CreERT2; R-spondin3^fl/fl^* mice, which enable acute chief cell injury by depletion of LGR5^+^ cells together with simultaneous conditional depletion of *Rspo3* expression in myofibroblasts. *LGR5-DTR-eGFP;MYH11-CreERT2;R-spondin3^WT/WT^* mice served as controls. Both mouse strains were treated with DT to deplete LGR5^+^ chief cells and with tamoxifen to deplete *Rspo3* expression in MYH11^+^ cells.

After a 7-day recovery period, LGR5^+^ cells reappeared in the gland bases in *LGR5-DTR-eGFP; MYH11-CreERT2; R-spondin3^WT/WT^* mice (Figure 3R), the GIF^+^ chief cell compartment appeared to be fully recovered (Figure 3T), and proliferative cells were again restricted to the isthmus region (Figure 3S). In contrast, in *LGR5-DTR-eGFP; MYH11-CreERT2; R-spondin3^fl/fl^* mice, LGR5 was not reexpressed (Figure 3U), fewer GIF^+^ cells were observed, and instead, GSII expression was present in the gland base, indicating inefficient chief cell recovery in mice lacking *Rspo3* (Figure 3, V and W). We quantified and compared the number of chief cells in DT-treated *LGR5DTR; Rspo3*-KO mice and DT-treated WT mice to that in nontreated control mice and found that the reduction in the number of chief cells upon loss of *Rspo3* expression could not be rescued by other signaling events upon chief cell depletion, indicating again the central role of RSPO3 for chief cell recovery (Supplemental Figure 4F). Of note, these results also demonstrated that RSPO3-driven chief cell regeneration could occur independently of *LGR5*^+^ chief cells. Furthermore, GIF^+^GSII^+^ cells that appeared in the gland base during regeneration were largely negative for Cd44v9, a marker for SPEM, suggesting that chief cell regeneration occurs through the proliferation of precursor cells and neighboring cells instead of through the proliferation of SPEM cells (Supplemental Figure 5B).

Since LGR5 is a receptor for RSPO3 and our previous data demonstrated that (re)expression of LGR5 is dependent on RSPO3 expression, we asked how gland injury or depletion of chief cells and subsequent induction of RSPO3 expression could affect in situ *LGR5* expression. To this end, we analyzed the expression pattern of *LGR5* in control mice, in mice sacrificed 1, 3, or 7 days after HDT treatment, and in LGR5DTR mice 1 day after LGR5^+^ cell depletion. We found that during homeostasis, *LGR5* expression was most abundant in the chief cell compartment, with additional low expression in the isthmus compartment. Upon HDT treatment, the expression of *LGR5* in the gland as well as in the isthmus region, which contains the proliferating cells during homeostasis, was markedly increased (Supplemental Figure 6A). Similarly, in LGR5DTR mice, despite DT-induced loss of LGR5^+^ cells, *LGR5* was expressed in the glandular compartment as well as in the isthmus region (Supplemental Figure 6B). Furthermore, no reduction in *LGR5* expression was detected by qPCR (Supplemental Figure 6C) upon depletion of LGR5^+^ cells.

Since LGR5 is a receptor for R-spondin, this raised the question of through which receptor R-spondin induced its effects upon depletion of LGR5^+^ cells. ISH for the homolog LGR4 in corpus sections of nontreated mice revealed that *Lgr4* was expressed throughout the gland (Supplemental Figure 6D). Therefore, we propose that, after the loss of LGR5^+^ cells, RSPO3 can affect the epithelium via binding to LGR4 receptors in the isthmus and gland, resulting in gland regeneration and recovery of LGR5^+^ chief cells. Of note, similarly to the homeostatic state, also upon injury, *Rspo3* expression was most abundant in the stroma beneath the gland base, with low levels of *Rspo3* expression in stromal cells adjacent to the isthmus (Supplemental Figure 7). In summary, we conclude that disruption of LGR5^+^ cell integrity is sufficient to cause a global change of proliferative kinetics in the gland and that epithelial regeneration and reestablishment of corpus gland homeostasis are orchestrated in an RSPO3-dependent manner.

### Glandular regeneration after HDT treatment and upon chief cell loss is mediated through YAP.

**Figure 4:**
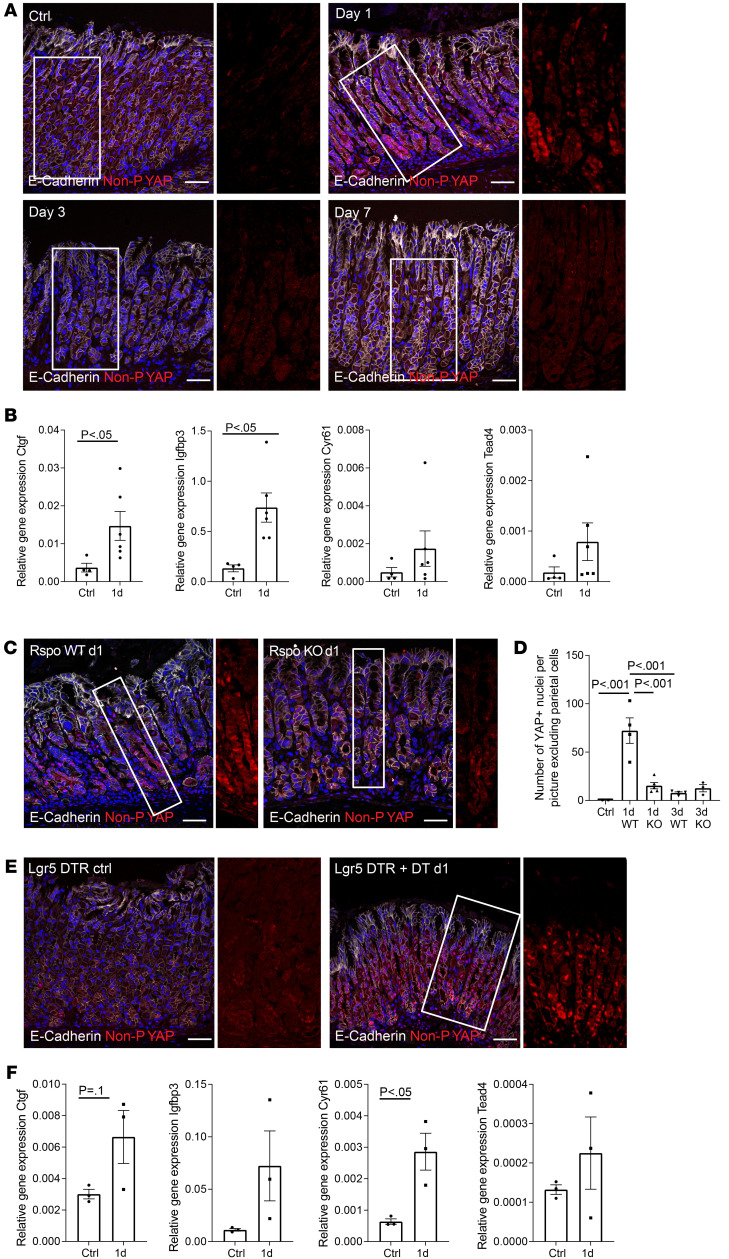
Glandular regeneration after HDT and upon chief cell loss is mediated through YAP. (**A**) Immunofluorescence costaining for nonphosphorylated YAP (red) and E-cadherin (white) in *Rspo3*-WT mice treated with HDT for 2 days and sacrificed on day 1, 3, or 7 after treatment and nontreated control. (**B**) qPCR data showing expression of YAP target genes *Ctgf*, *Igfbp3*, and *Cyr61* and the transcription factor *Tead4* in the corpus of *Rspo3*-WT mice treated with HDT for 2 days and sacrificed on day 1 (*n* = 6) versus nontreated controls (*n* = 4). (**C**) Immunofluorescence staining for nonphosphorylated YAP (red) and E-cadherin (white) in *Rspo3*-WT and *Rspo3*-KO mice treated with HDT for 2 days and sacrificed on day 1 after treatment. (**D**) Quantification of YAP^+^ nuclei per image excluding parietal cells in *Rspo3*-WT and *Rspo3*-KO mice treated with HDT for 2 days and sacrificed on day 1 or day 3 after treatment and nontreated control. (**E**) Immunofluorescence costaining for nonphosphorylated YAP (red) and E-cadherin (white) in LGR5DTR mice treated with DT on 3 consecutive days and sacrificed on day 1 after treatment and nontreated littermates. (**F**) qPCR data showing expression of YAP target genes *Ctgf,* *Igfbp3*, and *Cyr61* and the transcription factor *Tead4* in the corpus of LGR5DTR mice (*n* = 3) treated with DT on 3 consecutive days and sacrificed on day 1 after treatment versus nontreated littermate controls (*n* = 3). Scale bars: 50 μm. Unpaired parametric *t* test (**B** and **F**); 1-way ANOVA with Tukey’s multiple-comparison test (**D**).

Since proliferation and regeneration after gland injury were driven by upregulated RSPO3 expression, we asked how RSPO3 induces these effects. Both RSPO3 and yes-associated protein (YAP) signaling were recently shown to be important for colonic injury repair upon dextran sulfate sodium–induced (DSS-induced) colitis (12, 22), but their relationship has not been investigated. We hypothesized that proliferation upon acute injury is associated with RSPO3-induced YAP activation. To study this, we performed immunofluorescence staining of sections from *Rspo3*-WT mice sacrificed 1, 3, and 7 days after HDT treatment as well as from nontreated controls and found a large increase in the abundance of the active nonphosphorylated form of YAP protein 1 day after injury (Figure 4A), coinciding with the peak in *Rspo3* expression (see Figure 3C). Of note, expression of YAP was most abundant in the glandular compartment where hyperproliferation had also been observed. qPCR analysis for the YAP target genes *Ctgf*, *Igfbp3*, and *Cyr61* (23) as well as the transcription factor *Tead4* further confirmed an increase in YAP signaling 1 day after injury (Figure 4B). In the corpus of *Rspo3*-KO mice, qPCR analysis revealed lower gene expression for the target genes *Cyr61* and *Igfbp3* compared with that in WT mice, while *Ctgf* remained unchanged (Supplemental Figure 8, A–C). Since immunofluorescence staining revealed that YAP activation not only occurs in the epithelium, but also in stromal cells, leading to ambivalent qPCR results, we instead quantified the number of YAP^+^ epithelial nuclei (costained with E-cadherin) and found a significantly reduced number of YAP^+^ nuclei in mice lacking *Rspo3* expression (Figure 4, C and D) compared with WT mice. Similarly, immunofluorescence and qPCR analysis on sections of LGR5DTR mice at 24 hours after DT-induced depletion of chief cells confirmed activation of YAP signaling that coincided with the upregulation of *Rspo3* expression (Figure 4, E and F), indicating a central role of RSPO3 for activation of YAP and proliferation of gland cells upon injury.

### RSPO-driven YAP expression is a prerequisite for proliferation in organoids.

**Figure 5:**
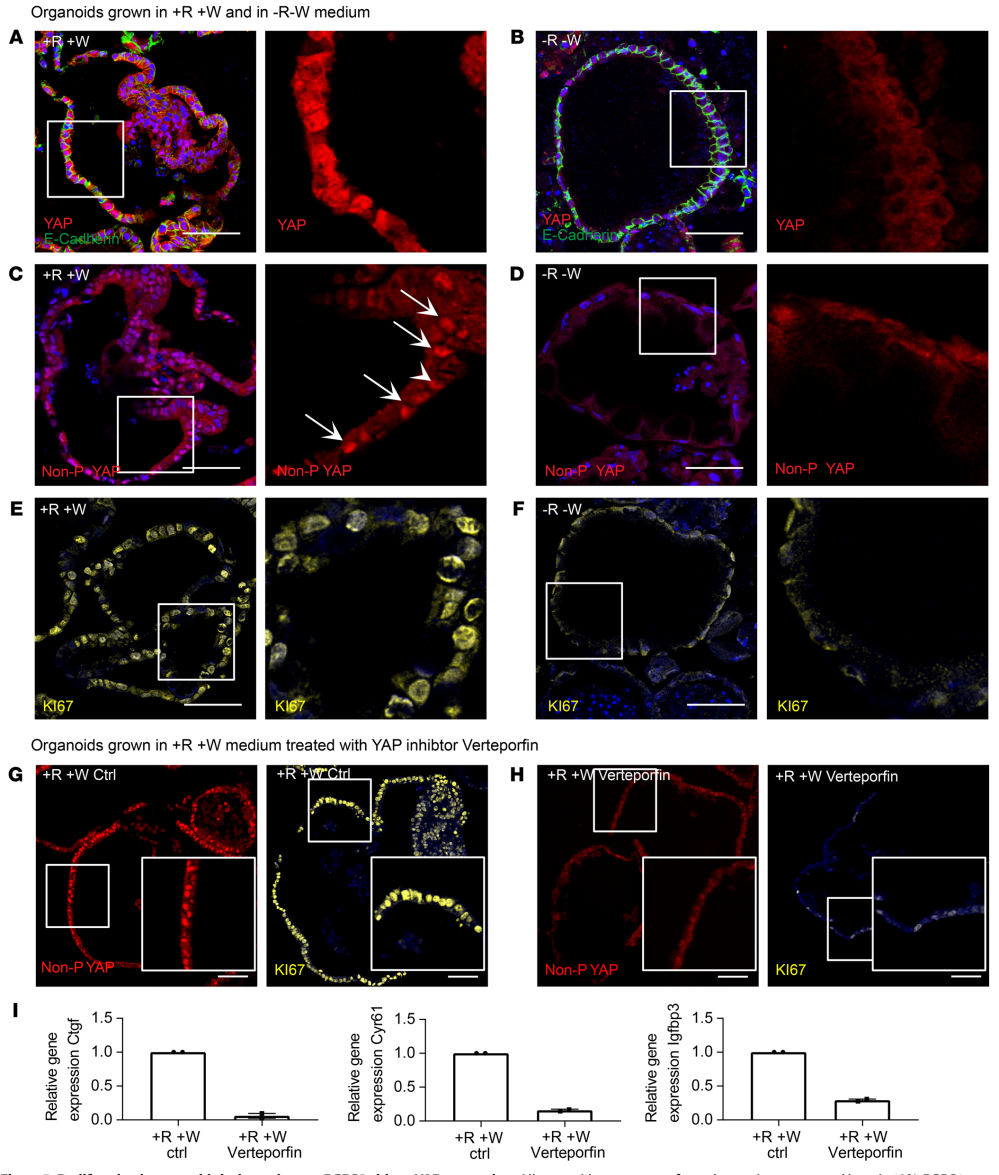
Proliferation in organoids is dependent on RSPO3-driven YAP expression. All organoids were grown from the murine corpus and kept in 10% RSPO1 conditioned medium. (**A** and **B**) Representative immunofluorescence images of costaining for YAP (red) and E-cadherin (green) in sections from organoids grown in (**A**) +R+W and (**B**) –R–W media. (**C** and **D**) Representative immunofluorescence images of staining for nonphosphorylated (active nuclear) YAP (red) in sections from organoids grown in (**C**) +R+W and (**D**) –R–W media. (**E** and **F**) Representative immunofluorescence images of costaining for Ki67 (yellow) in sections from organoids grown in (**E**) +R+W and (**F**) –R–W media. Arrows highlight YAP-positive nuclei. (**G**) Representative immunofluorescence images of staining for nonphosphorylated (active nuclear) YAP (red) and Ki67 (yellow) in sections from organoids grown in full medium. (**H**) Representative immunofluorescence images of staining for nonphosphorylated (active nuclear) YAP (red) and Ki67 (yellow) of sections from organoids grown in full medium and treated with verteporfin for 24 hours. (**I**) qPCR data showing expression of YAP target genes *Ctgf*, *Cyr61,* and *Igfbp3* of organoids grown in full medium and treated with verteporfin for 24 hours and nontreated control organoids (*n* = 2 per group). Scale bars: 50 μm.

To further explore the role of R-spondin in YAP signaling, we grew murine organoids in either +R+W or –R–W medium. Organoids cultured in full medium exhibited high levels of total YAP protein (Figure 5A), while≈removal of R-spondin/WNT induced an extensive loss of YAP protein expression (Figure 5B), indicating that R-spondin/WNT signaling is an important prerequisite for YAP signaling. Staining for nonphosphorylated YAP revealed that, in contrast with organoids grown without WNT and R-spondin (Figure 5D), organoids grown in +R+W medium showed high levels of nuclear YAP (Figure 5C). Similarly, organoids grown in +R+W medium were also positive for Ki67 (Figure 5E), while those grown in –R–W medium were not (Figure 5F). Indeed, active YAP signaling, as well as Ki67-positive nuclei, were detected in GIF^+^GSII^+^ as well as GIF^–^GSII^–^ cells, indicating that organoids grown in +R+W medium do not fully resemble the homeostatic chief cell state, but instead are at least in part representative of the regenerative state (Supplemental Figure 9A).To evaluate whether YAP signaling is crucial for organoid proliferation, we grew organoids in +R+W medium for 5 days and then treated them for 24 hours with the YAP inhibitor verteporfin, which interferes with the binding of YAP to the transcription factor TEA domain family member (TEAD). Since *YAP* expression during the first 2 days after passage is required for establishing intestinal organoid culture (24), we used the inhibitor at the later time point of 5 days after seeding, when cells also start expressing markers of differentiated cell types. qPCR for the YAP target genes *Ctgf*, *Igfbp3*, and *Cyr61* confirmed a significant reduction of YAP signaling upon verteporfin treatment (Figure 5I), and immunofluorescence staining for nonphosphorylated YAP further confirmed the reduction of YAP activity after treatment (Figure 5, G and H). Finally, immunofluorescence staining for Ki67 showed that proliferation was indeed strongly inhibited upon treatment (Figure 5, G and H).

To verify the results obtained with verteporfin, we used 2 additional experimental setups. First, we grew murine organoids from single cells and treated them on day 2 with the Src inhibitor PP2, which is involved in YAP signal transduction. Two days after treatment, quantification of organoid size revealed significant inhibition of organoid expansion compared with nontreated organoids (Supplemental Figure 9, B and C). Second, we grew organoids from the corpus of Yap/Taz^fl/fl^ mice. Organoids were split into single cells and treated with TAT-Cre recombinase to induce KO. Two days after induction, organoid formation efficiency was quantified and compared with that in corresponding nontreated cultures. The toxicity of TAT-Cre recombinase was measured by adding the protein to C57BL/6 (Bl6) organoid cultures, which led to a 40% reduction in organoid-forming capacity. In contrast, the organoid formation capacity of Yap/Taz^fl/fl^ organoids was reduced by 80%, indicating that Yap/Taz does indeed play a crucial role in the proliferation of corpus cells (Supplemental Figure 9, D and E). Together, these results suggest that YAP is induced by R-spondin signaling and that its activation mediates epithelial proliferation in the corpus.

### Human ulcers show high stromal expression of RSPO3 in the ulcer bed and active YAP signaling in adjacent epithelial cells.

Next, we asked whether the regenerative response observed in mice upon epithelial injury is also activated in human pathologies. To this end, we analyzed the spatial expression pattern of *RSPO3* and active YAP protein in human ulcer samples. ISH for *RSPO3* revealed a strong expression of *RSPO3* in stromal cells in the ulcer bed (Supplemental Figure 10). Further immunofluorescence staining for active YAP and Ki67 revealed that, in parallel with our findings in murine tissue, human ulcer epithelial cells adjacent to the injury site showed strong activation of YAP that was accompanied by intense proliferation (Supplemental Figure 10), indicating a common response to injury.

### RSPO3 causes glandular proliferation upon H. pylori infection.

*H*. *pylori* is a bacterial pathogen that colonizes the stomach, causes chronic gastritis, and increases the risk of gastric cancer (25–27). In mice, *H*. *pylori* causes mucosal inflammation and alterations in gastric gland homeostasis. Changes in the gland base characterized by accumulation of GIF^+^GSII^+^ cells are frequently observed upon *H*. *pylori* infection and are considered a premalignant metaplastic feature, called SPEM (6–9).

**Figure 6:**
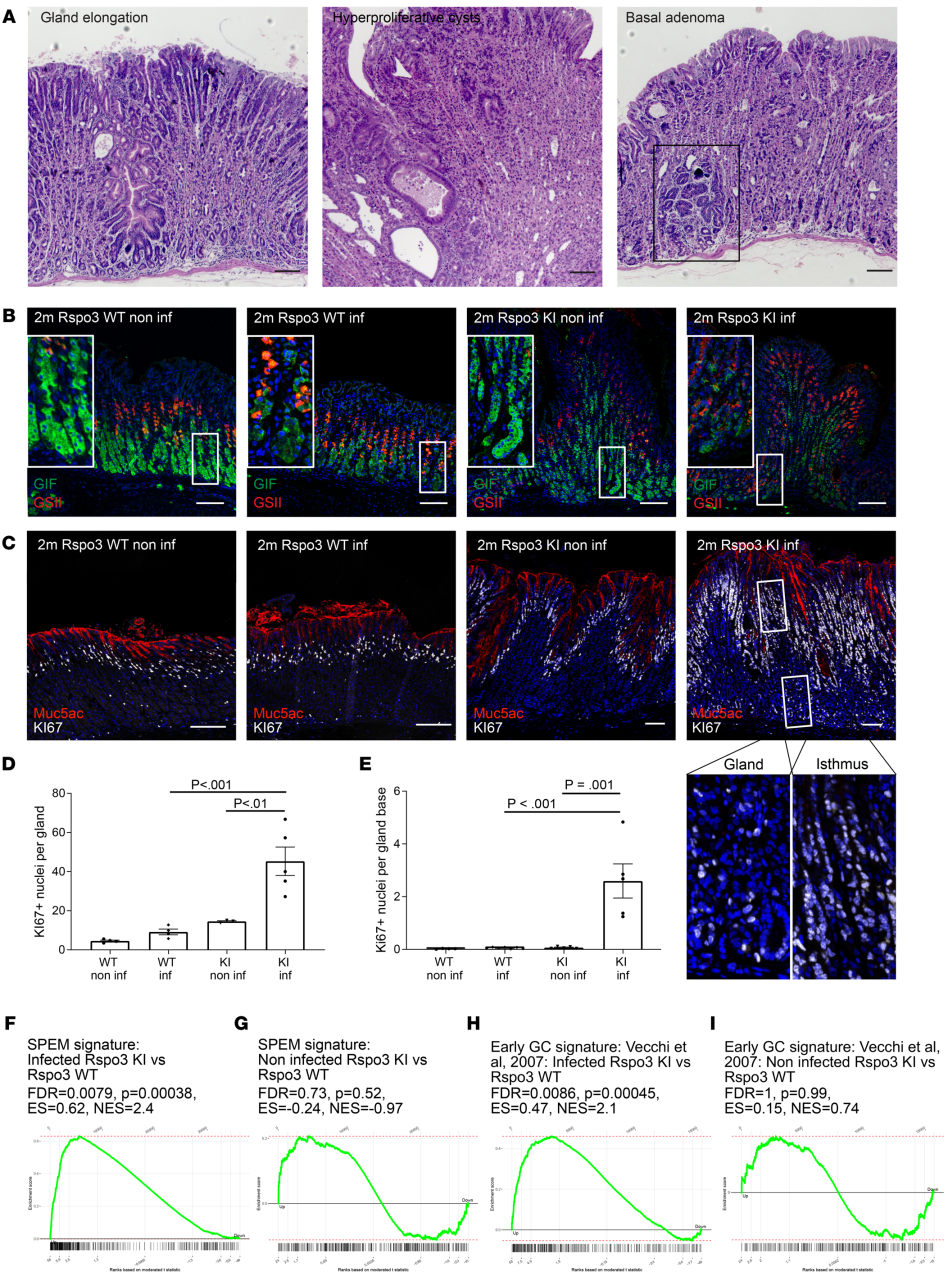
RSPO3 causes glandular proliferation upon *H. pylori* infection. (**A**) H&E staining of 2-month–infected *Rspo3*-KI mice. (**B**) Representative immunofluorescence images of costaining for GSII (red) and GIF (green) of sections from noninfected and 2-month–infected *Rspo3*-WT and *Rspo3*-KI mice. (**C**) Representative immunofluorescence images of Ki67 staining (white) and MUC5AC (red) of sections from noninfected and 2-month infected *Rspo3*-WT and *Rspo3*-KI mice. (**D**) Quantification of Ki67^+^ cells per gland of infected *Rspo3*-WT (*n* = 4 mice) and *Rspo3*-KI mice (*n* = 5) and noninfected littermate controls (*n* = 3–4). (**E**) Quantification of proliferating cells per gland base of infected *Rspo3*-WT (*n* = 7) and *Rspo3*-KI mice (*n* = 5) and noninfected littermate controls (*n* = 4–5). (**F** and **H**) GSEA of microarray data comparing the expression profile of the corpus tissue from 2-month–infected *Rspo3*-KI mice and infected littermate controls treated with tamoxifen 2 months before euthanasia with a published data set for (**F**) SPEM (28). (**H**) Early gastric cancer (GC) signature (29). (**G** and **I**) GSEA of microarray data comparing the expression profile of the corpus tissue from noninfected *Rspo3*-KI mice and noninfected littermate controls treated with tamoxifen 14 days before euthanasia with a published data set for (**G**) SPEM (28). (**I**) Early gastric cancer signature (29). ES, enrichment score; NES, normalized enrichment score. For GSEA, *n* = 2 mice per group. Scale bars: 100 μm. One-way ANOVA with Tukey’s multiple-comparison test.

Since RSPO3 drove chief cell differentiation and recovery, we asked whether elevated RSPO3 expression could prevent the corpus gland alterations normally seen in the context of chronic *H*. *pylori* infection. Therefore, we infected WT as well as *Rspo3*-KO and *Rspo3*-KI mice with *H*. *pylori* for 2 months. As expected, immunofluorescence labeling revealed that, upon infection of *Rspo3*-WT mice with *H*. *pylori,* the GIF^+^ compartment was reduced and the GIF^+^GSII^+^ cell compartment was enlarged (Supplemental Figure 11, A–D). In noninfected *Rspo3-*KO mice, the GIF^+^ chief cell compartment was reduced and the number of cells per gland coexpressing GIF^+^GSII^+^ was increased. Infection of *Rspo3*-KO mice led neither to a further reduction of the GIF compartment nor to a further increase of GIF^+^GSII^+^ cells per gland in *Rspo*3-KO mice (Supplemental Figure 11, A–D). The number of parietal cells was significantly affected neither by a reduction of *Rspo3* expression nor by infection of *Rspo3*-WT mice with *H*. *pylori* or a combination of *Rspo3*-KO and *H*. *pylori* infection (Supplemental Figure 11E). Surprisingly, infected *Rspo3*-KI mice showed dramatic changes in gland architecture, such as extreme gland elongation, formation of large cyst-like structures, and in 30% of mice, development of focal lesions in the base of corpus glands that were classified as adenoma by a veterinarian expert (Figure 6A and Supplemental Figure 12A).

Immunolabeling and quantitative analysis revealed that, similarly to those of infected WT mice, the glands of infected KI mice harbored GIF^+^ cells that coexpressed GSII — a characteristic sign of SPEM (Figure 6B). However, in *Rspo3*-KI mice, this GIF^+^GSII^+^ compartment was significantly enlarged compared with that in infected WT mice (Figure 6B and Supplemental Figure 12, B–D). Again, the number of parietal cells was not affected by infection with *H*. *pylori*, but their number was increased in *Rspo3*-overexpressing mice compared with controls (Supplemental Figure 12E).

We next assessed the proliferation in the different mice using immunolabeling for Ki67 (Figure 6C and Supplemental Figure 11A). Infection triggered a slight increase in Ki67^+^ cells in WT mice (Figure 6, C and D). In contrast, we observed massive hyperproliferation in *Rspo3*-KI mice upon infection (Figure 6, C and D). Of note, in contrast to what occurred in uninfected *Rspo3*-KI mice and infected WT mice, proliferation was not restricted to the isthmus, but was also prominent in the glandular compartment (Figure 6E). To characterize this in more detail, we performed a transcriptome analysis from the corpus of 2-month–infected *Rspo3*-KI and *Rspo3*-KO mice versus corresponding infected WT littermate controls. GSEA demonstrated that several genes involved in DNA replication were downregulated in infected *Rspo3*-KO mice compared with in WT mice and were upregulated in infected *Rspo3*-KI mice compared with in WT infected littermates (Supplemental Figure 11F and Supplemental Figure 13A). Furthermore, the “SPEM” gene set (28) as well as the “gastric cancer” gene set (29) showed significant negative enrichment in infected *Rspo3*-KO mice compared with WT and positive enrichment in infected *Rspo3*-KI mice compared with WT littermates (Supplemental Figure 11G and Figure 6, F and H).

When we performed the same analysis comparing the expression pattern in uninfected *Rspo3*-KI mice versus uninfected controls, we did not observe a significant enrichment for those gene sets (Supplemental Figure 13A and Figure 6, G and I). Additional immunofluorescence labeling for the SPEM marker CD44v9 confirmed strong labeling of base cells in infected *Rspo3*-overexpressing mice and lower signal in infected *Rspo3*-WT mice, but no signal in noninfected *Rspo3-*overexpressing or WT mice (Supplemental Figure 13B), further indicating a regulatory circuitry of RSPO3 and *H*. *pylori* infection. In addition, in human ulcers, which typically coincide with *H*. *pylori* infection, positive staining for CD44v9 was also detected at the ulcer margin together with strong RSPO3 expression, but was absent from the epithelium more distant from the ulcer site (Supplemental Figure 13C).

Importantly, the stronger response to infection by *Rspo3*-overexpressing mice compared with WT mice was not driven by higher bacterial counts (Supplemental Figure 12F) or an additional upregulation of *Rspo3* upon infection (Supplemental Figure 12G). Instead, an upregulation of gene expression of the antimicrobial proteins Reg3b, Reg3g, Cd74, and Itln1 was observed in infected *Rspo3-*overexpressing mice compared with infected WT mice (Supplemental Figure 12H), which parallels recent observations in the stomach antrum (11).

### Gland cells express YAP, which is activated upon infection with H. pylori.

**Figure 7:**
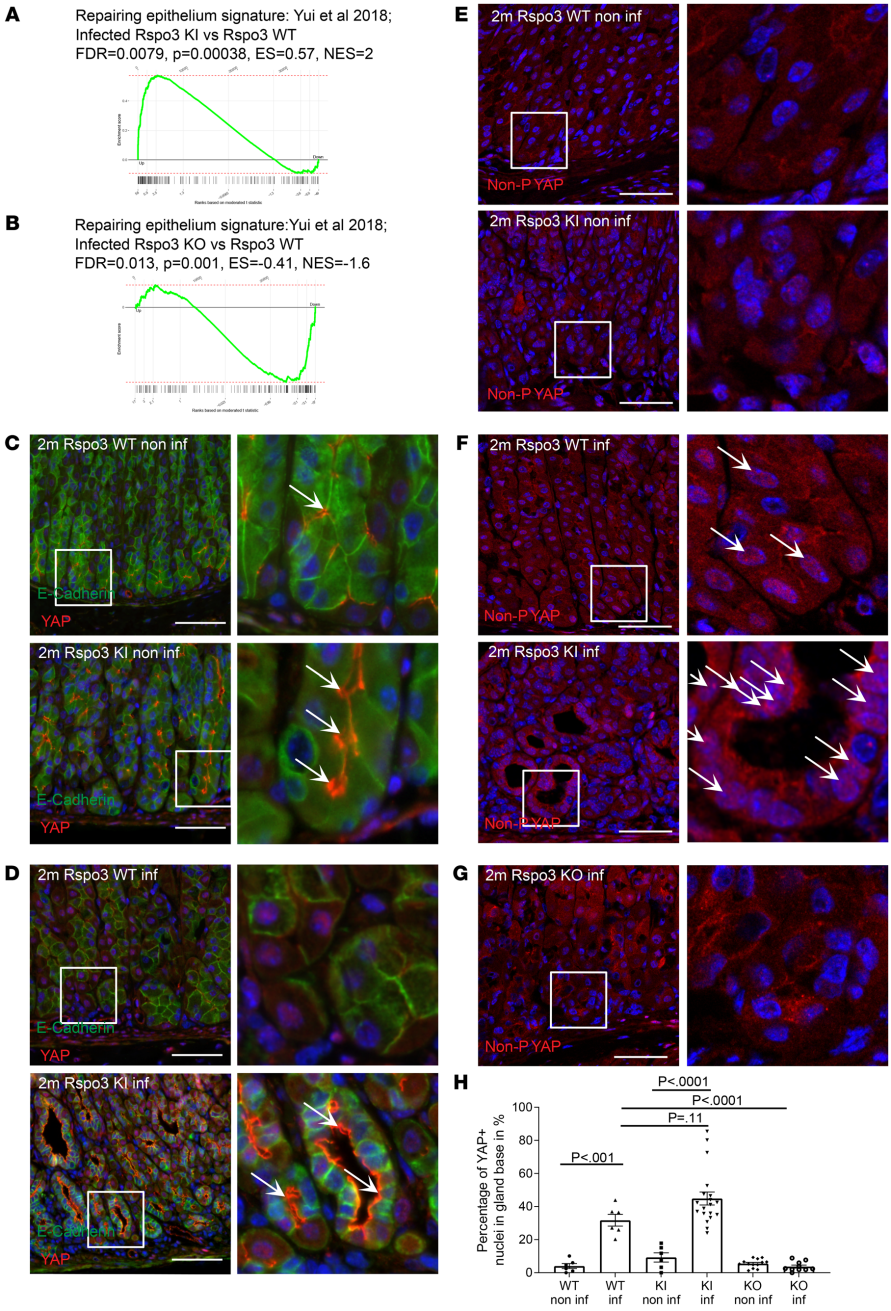
Gland cells express YAP, which upon infection with *H. pylori*, is translocated to the nucleus. (**A** and **B**) GSEA of microarray data comparing the expression profile of the corpus tissue from (**A**) 2-month–infected *Rspo3*-KI mice and infected littermate controls treated with tamoxifen 2 months before euthanasia, (**B**) 2-month–infected *Rspo3*-KO mice and infected littermate controls treated with tamoxifen 2 months before euthanasia with a published data set for repairing epithelium signature (22). (**C** and **D**) Representative immunofluorescence images of costaining for YAP (red) and E-cadherin (green) of sections from (**C**) noninfected and (**D**) infected *Rspo3*-KI and *Rspo3*-WT mice. Arrows show cytoplasmatic YAP. (**E**–**G**) Representative immunofluorescence images of nonphosphorylated (=active nuclear) YAP (red) of sections from (**E**) noninfected, (**F**) infected *Rspo3*-WT, and *Rspo3*-KI mice, and (**G**) infected *Rspo3*-KO mice. (**H**) Percentage of epithelial YAP^+^ nuclei (costained with E-cadherin) in the gland bases of sections from noninfected and 2-month–infected *Rspo3*-KI, *Rspo3*-KO, and *Rspo3*-WT mice. For GSEA, *n* = 2 mice per group. Scale bars: 50 μm. One-way ANOVA with Tukey’s multiple-comparison test.

We hypothesized that RSPO3-dependent glandular proliferation in the context of *H*. *pylori* infection is also mediated by increased YAP activation. We performed GSEA with a gene set from colonic epithelial cells undergoing regeneration upon DSS-induced colitis, which is driven by YAP signaling (22). We confirmed a positive enrichment of genes upregulated in intestinal epithelial cells undergoing regeneration (22) in infected *Rspo3*-KI mice compared with infected WT mice (Figure 7A) and downregulation of those genes in infected *Rspo3*-KO mice compared with infected WT mice (Figure 7B).

Immunofluorescence labeling for YAP in noninfected WT mice showed that, even under homeostatic conditions, gland cells express membrane-associated YAP (Figure 7C). Mice that overexpress *Rspo3* and accumulate gland cells also express higher levels of YAP in the gland, which is again mainly localized to the membrane (Figure 7C). Upon infection, we noticed a translocation of YAP to the nucleus (Figure 7D).

To assess YAP activation, we performed immunofluorescence labeling for nonphosphorylated YAP, the active, nuclear form. We confirmed that infection with *H*. *pylori* induces a shift of predominantly membrane-associated to predominantly nuclear YAP and that the active form of the protein is also more abundant in infected *Rspo3*-KI mice compared with infected WT mice (Figure 7, E and F). Furthermore, the YAP staining colocalized with GSII^+^ cells, including the gland base in infected mice (Supplemental Figure 14).

In contrast, in infected *Rspo3*-KO mice, YAP protein was rarely found in the nucleus (Figure 7G). Nuclear YAP was also absent in WT mice infected with a type IV secretion system–deficient *H*. *pylori* strain (isogenic ΔCagE mutant) (Supplemental Figure 15, A and B)and those mice also failed to show coexpression of GIF^+^ and GSII^+^, indicating that the induction of a regenerative response, including YAP activation, depends on the type IV secretion system (Supplemental Figure 15, C and D). For quantitative analysis, we assessed the percentage of YAP^+^ nuclei of all nuclei of E-cadherin–positive cells in the lower part of the gland, where the signal was absent in homeostatic conditions, confirming active YAP signaling upon infection in the presence of RSPO3 signaling (Figure 7H), with enhanced signaling upon *Rspo3* overexpression, and reduced active YAP signaling in mice that lack *Rspo3* expression.

In summary, we find that RSPO3 induces YAP expression in gland cells and that infection with *H*. *pylori* promotes activation of the YAP signaling pathway.

## Discussion

Stromal RSPO3 is known to induce stem cell turnover in different systems (10, 11, 30). However, our data provide evidence that it can also be a decisive signal to orchestrate differentiation toward specific lineages, thereby controlling the number of differentiated cells in a tissue. Specifically, we discovered that in the corpus, RSPO3 promoted differentiation into glandular secretory lineages toward parietal and chief cells at the expense of the pit lineage.

Furthermore, we revealed that loss of RSPO3-dependent secretory gland cells triggered an increased expression of RSPO3 that in turn orchestrated their regeneration and recovery. Moreover, this RSPO3-driven regenerative response is associated with activation of YAP signaling and the development of premalignant gastric lesions observed upon *H*. *pylori* infection. While RSPO3 expression shows a short-term peak immediately after acute loss of parietal and chief cells and promotes their recovery under these conditions, the prolonged overexpression of RSPO3 in the context of *H*. *pylori* infection “locks” the epithelium in a regenerative state that serves as the basis for severe hyperplasia and premalignant metaplasia.

There is an ongoing debate about the cell of origin of malignant as well as premalignant lesions in the corpus. Chief cells expressing LGR5 have been extensively studied in this context. After it was demonstrated that LGR5^+^ cells have the capacity to promote gastric carcinogenesis upon mutation of Kras (16), other studies, including a recent one using *Gpr30* as a chief cell marker, concluded that chief cells are unlikely to give rise to metaplasia and cancer lesions (31). Yet another recent lineage-tracing study using a new GIF-Cre-RnTnG model did observe GIF^+^ cell–derived SPEM lesions (32). Our data indicate that injury of the gland causes a global proliferative response that involves different cells, suggesting that it is likely that more than one specific cell type is capable of repopulating injured glands. Importantly, we show that depletion of LGR5^+^ cells is sufficient to cause such regenerative responses. Our study demonstrates that cellular differentiation and expression of cell type–specific genes are controlled by niche signals. Consequently, changes in these signals, such as elevated expression of RSPO3, can rapidly alter the glandular cell states. This enables high plasticity and rapid cellular adaptation while being a challenge for the interpretation of lineage-tracing studies.

While we find that high expression of RSPO3 in the stroma can result from injury and infection, elevated R-spondin signaling can also occur as a result of a mutational event in genes such as *RNF43*, which is observed in a subset of patients with gastric cancer (33). Our results here demonstrate how such mutations may predispose to but are not necessarily sufficient to induce carcinogenesis — which may require an additional trigger in the form of cell loss, such as that driven by *H*. *pylori* infection. Accordingly, *H*. *pylori* have been shown to aggravate gastric pathology in a mouse model of *Rnf43* mutation (34).

Of note, we observed that in the context of epithelial regeneration, RSPO3-driven effects were not limited to the basal gland compartment immediately above the RSPO3-expressing fibroblasts, but that changes in differentiation and proliferation were also induced in the isthmus and mucous cell compartments, while RSPO3 expression was still mainly restricted to the subglandular compartment, indicating that even a small amount of R-spondin can affect gland cells that are not in direct proximity to RSPO3-expressing cells. We have recently reported that in the antrum, in addition to RSPO3, BMP signaling also forms a gradient along the base-lumen axis and affects epithelial differentiation and that BMP signaling was altered upon infection with *H*. *pylori* (35). Thus, in addition to an absolute increase in RSPO3 expression, further changes in niche signaling may affect the responsiveness of cells in the context of infection and epithelial injury.

We find that glandular regeneration is characterized by transcriptional activity of YAP and that RSPO3 signaling is required for the expression of YAP. The YAP-driven regenerative epithelial state (36) has also been observed upon intestinal injury by ionizing radiation (36) and upon DSS-induced colitis in mice (22). Both types of injury trigger a loss of LGR5^+^ stem cells, followed by reprogramming of more differentiated cells that reenter the cell cycle. In this context, R-spondin has also been shown to be upregulated and to induce epithelial reprogramming (12). Our data here suggest that RSPO3 is responsible for maintaining high levels of YAP expression, which enables tissue regeneration via rapid activation of YAP signaling and subsequent cellular proliferation. The exact mechanism of YAP activation is not clear, but previous studies identified mechanical signals as a trigger of YAP activation (37, 38) and our findings of YAP activation upon disruption of epithelial integrity via loss of chief cells parallel this concept. Further studies revealed that activation of ERK signaling (39), mTOR signaling (40), and IL-33 pathway (41) as well as cystine-dependent ROS neutralization (42) are involved in the development of SPEM. Indeed, our transcriptome data from *H*. *pylori*–infected mice overexpressing RSPO3 show activation of mTOR signaling and higher expression of IL-33, suggesting that these factors are involved in the activation of YAP upon injury.

In summary, we find that stromal signals such as RSPO3 play a critical role in orchestrating physiological epithelial regeneration as well as the development of epithelial premalignant lesions. Therefore, further investigations on the interplay between epithelial and stromal signaling pathways, as well as between different stromal and different epithelial signaling pathways, respectively, will likely provide further critical insights into the processes that drive epithelial homeostasis and carcinogenesis. Finally, those findings will provide avenues for therapeutic interventions for patients with high-risk gastric lesions.

## Methods

### Mouse experiments.

All animals were maintained in autoclaved microisolator cages and provided with sterile drinking water and chow ad libitum. *LGR5–eGFP–IRES-CreERT2* mice (43) were purchased from the Jackson Laboratory, and *LGR5DTR-eGFP* (44) mice were provided by Genentech. Rspo^fl/fl^ mice (45) were a gift from J. Cobb (University of Calgary, Calgary, Canada). *MYH11CreERT2* (46) mice were provided by S. Offermanns (Max Planck Institute for Heart and Lung Research, Bad Nauheim, Germany). To generate conditional KO mice with depletion of *Rspo3* in myofibroblasts, we bred *Rspo3^fl/fl^* mice with *MYH11-CreERT2* mice, while *MYH11-CreERT2* littermates bred with WT *Rspo3* alleles served as controls. To further deplete LGR5^+^ cells in these mice, they were bred with *LGR5-DTR-eGFP* mice. Mice that conditionally overexpress *Rspo3* under the *Rosa26* promoter (*Rosa26Sor6[Cag-Rspo3]*) were described previously (15). These animals were bred with *MYH11-CreERT2* mice to generate double-heterozygous *MYH11-CreERT2/Rosa26Sor6(CAG–Rspo3)* mice.

Six- to eight-week-old male mice were used for this study. Littermates were used for the experiments and randomly allocated to experimental groups. To induce KO or KI of *Rspo3*, tamoxifen (MilliporeSigma) was injected intraperitoneally as a single dose (4 mg/25 g body weight, diluted in 200 μl corn oil) at the indicated time points. For short-term corpus injury, mice were first treated with a single induction dose of 4 mg per 25 g body weight tamoxifen to induce *Rspo3* KO, were allowed to recover for 14 days, and were then injected with HDT (5 mg per 20 g body weight, diluted in 200 μl corn oil) on 2 consecutive days (20, 21).

DT (MilliporeSigma, 50 μg per kg/1.25 μg per 25 g body weight) was injected intraperitoneally on 3 consecutive days. For recovery analysis upon loss of LGR5^+^ cells, *LGR5-DTR-eGFP; MYH11-CreERT2; Rspo3^fl/fl^* mice were treated with 1 dose of tamoxifen and 1 dose of DT on the same day. At the time of harvest, the forestomach was removed, and the glandular stomach was opened along the lesser curvature and laid flat. The stomach contents were removed, and the tissue was divided into 2 halves along the greater curvature. For microscopy analysis, a similar longitudinal section at the midline along the greater curvature was used in all animals to minimize sampling error. Experiments were performed in at least 3 biological replicates per condition. For all experiments, mice were randomly allocated to experimental groups.

### H. pylori infection.

Mice were orally infected with a single dose of 10^8^ *H*. *pylori* PMSS1 or isogenic deletion mutants of CagE (47) and were euthanized at indicated time points. A longitudinal section of stomach tissue was weighed and then mechanically homogenized in brain-heart infusion medium. Serial dilutions of the homogenates were plated for enumeration of colony-forming units, and bacterial counts were expressed as colony-forming units per gram of stomach to ensure infection efficiency. Experiments were performed in at least 3 biological replicates per condition. Mice were randomly allocated to experimental groups.

### Confocal microscopy.

The tissue was fixed in 4% paraformaldehyde for 2 hours and washed 2 times with PBS. Tissue was embedded in 4% agarose, and longitudinal stomach sections 300 μm thick were generated using a vibratome (Leica). Sections were permeabilized in PBS with 3% BSA, 1% saponin, and 1% Triton X-100 before staining. The samples were counterstained overnight with DAPI (MilliporeSigma) to visualize the nuclei and with Alexa Fluor 647 fluorophore–conjugated phalloidin (Life Technologies, A22287) to visualize cell boundaries. Samples were imaged with a Leica Sp8 confocal microscope.

### Agarose sections for LGR5eGFP^+^ cell imaging.

Longitudinal sections of mouse stomach were fixed in 4% PFA for 2 hours and washed 2 times with PBS to preserve immunofluorescence. Samples were embedded in 4% agarose and 300 μm sections cut using a vibratome (Leica). Sections were stained for 2 hours with DAPI in blocking buffer (1× PBS, 3% BSA, 1% saponin, 2% Triton X-100, 0.02% sodium azide).

### Histopathology.

Longitudinal sections of the stomach were fixed in 4% PFA overnight. Samples were paraffin embedded, sectioned, and stained with H&E as well as with anti-CD3 antibody by the Charité Core Unit Immunopathology for Experimental Models. Images were acquired using a Leica DMR microscope with ×10 and ×40 lenses.

### Immunofluorescence.

Paraffin-embedded sections were rehydrated and, following an antigen-retrieval step, incubated overnight with the following primary antibodies: rabbit anti-Ki67 (1:100, catalog D3B5, Cell Signaling Technology), FITC-conjugated rat anti-Ki67 (1:100, Thermo Fisher, catalog 11-5698-82), Alexa Fluor 647–conjugated GSII lectin (1:400, Thermo Scientific), rabbit anti-GIF (1:400, gift from David Alpers, Washington University School of Medicine, St. Louis, Missouri, USA), mouse anti-MUC5AC (1:100, Invitrogen, catalog 12178), sheep anti-pepsinogen II/PGC (1:100, Abcam, catalog ab9013), mouse anti–H-K-ATPase (1:500; MBL International Corp., catalog D032-3), mouse anti–E-cadherin (1:300, BD Biosciences, catalog 610181), rabbit anti-YAP (1:100, Cell Signaling Technology, catalog D8H1X, PE conjugated), rabbit anti-active YAP1 (1:100, Abcam, catalog 205270), and rat anti-CD44 v10-e16 (clone RM1, LKG-M002, Cosmo Bio Co.). Overnight incubation was followed by a washing step and incubation with the following secondary antibodies for 1 to 2 hours: donkey anti-rabbit Cy3 (Dianova, catalog 711-166-152, lot 115281), donkey anti-sheep Cy2 (Dianova, catalog 713-225-003, lot 88209), donkey anti-mouse Alexa Fluor 647 (catalog 715-605-150, lot 155989), goat anti-rat 488 (Dianova, catalog 112-546-062, lot 133084), and goat anti-rabbit Cy3 (Dianova, catalog 115-165-146, lot 109201). Tissue sections were counterstained with DAPI for nucleus visualization. For each secondary antibody, a control using only the secondary antibody was performed. Samples were imaged with a Leica Sp8 confocal microscope. Tile scans of human samples were imaged with a Zeiss Observer 7 microscope.

To quantify the respective size and location of the GIF-, PGC-, GSII-, and Ki67-positive gland compartments, each gland was measured for its height, the distance from gland base to first positive cell, height of the positive gland compartment, and distance from most apical positive cell to the top of the gland. The ratio between the distances was defined as the relative size of the respective gland compartment. Additionally, for analysis of the proliferative compartment, the number of Ki67-positive nuclei per gland were counted. All vertically cut corpus glands from a longitudinal section through the mouse stomach were used for the analysis. The investigator was blinded for image analysis.

### Single-molecule RNA ISH.

Tissue sections cut at 5 μm thickness were processed for RNA in situ detection using an RNAscope Red Detection Kit according to the manufacturer’s instructions (Advanced Cell Diagnostics). RNAscope probes against mouse *Rspo3* (402011, lot 16321A), mouse *LGR5* (312171, lot 21214A), and human *RSPO3* (491461, Lot 18137A) were used; positive and negative control probes were run in parallel and used according to the manufacturer’s instructions. Images were acquired using a Zeiss Observer 7.

### Organoid cultures.

Mouse strains used for organoid cultures were Bl6 and Yap/Taz^fl/fl^. Yap/Taz^fl/fl^ mice were a gift from H. Gerhardt (Max Delbrück Center for Molecular Medicine).

Corpus tissue was dissected from the mouse stomach and immediately incubated for 15 minutes in 0.04% sodium hypochlorite, followed by incubation for 90 minutes in a buffered saline solution containing 0.5 mM DTT and 3 mM EDTA to dissociate gastric glands. Glands were mechanically isolated from the tissue, and isolated glands were washed with PBS. One hundred glands were mixed with 30 μl Matrigel (BD) per well and plated in 24-well plates. After polymerization of Matrigel, murine gastric culture medium (advanced DMEM/F12 supplemented with B-27, N-2 [Invitrogen], *N*-acetyl cysteine [MilliporeSigma], penicillin/streptomycin, and containing 50 ng ml^−1^ epidermal growth factor, 100 ng ml^−1^ Noggin, 100 ng ml^−1^ fibroblast growth factor 10, 10 mM gastrin, RSPO1-conditioned medium [or 250 ng ml^−1^ human recombinant Rspo3] and WNT3A-conditioned medium) was added to the well surrounding the gland-containing Matrigel drop. The medium supplemented with growth factors was replaced every 2 to 3 days. After the first passage, organoids were grown for 2 days in full medium and then exposed to different concentrations of WNT and R-spondin–conditioned medium. As well as full medium, different concentrations of R-spondin–conditioned medium were added to the cultures for 5 days. Organoids were harvested on day 7. For verteporfin treatment, organoids were grown in full medium for 5 days, then treated with 1.25 nM verteporfin (MilliporeSigma, SML0534-MG) for 24 hours and harvested afterward. For treatment with the Src inhibitor PP2, organoids were grown in full medium for 2 days, then treated with 10 μM PP2 and harvested 2 days later.

To grow Yap/Taz^fl/fl^ organoids, corpus glands were isolated from Yap/Taz ^fl/fl^ mice and grown for 1 passage. Then organoids were split into single cells and kept as a cell suspension in full medium supplemented with 5 μM TAT-Cre recombinase. Single cells were incubated at 37°C, 5% CO_2_ for 2 hours to induce the P-lox system. Cells were collected, washed with PBS, and seeded into Matrigel. For paraffin embedding, organoids were fixed in 3.7% formaldehyde for 3 hours, washed twice with 0.1% BSA in PBS, and embedded in agarose. Samples were then manually dehydrated, paraffinized, and cut into 4 μm thick sections.

### Microarray analysis.

For microarray analysis, *Rspo3-*WT and *Rspo3*-KI mice were treated with tamoxifen 14 days before euthanasia. The corpus was removed and total RNA was isolated with TRIzol (Life Technologies) according to the supplier’s protocol using glycogen as coprecipitant. Microarray experiments were performed as independent dual-color dye-reversal color-swap hybridizations using 2 biological replicates. Quality control and quantification of total RNA were assessed using an Agilent 2100 Bioanalyzer (Agilent Technologies) and a NanoDrop 1000 UV-Vis spectrophotometer (Kisker). RNA labeling was performed with a dual-color Quick-Amp Labeling Kit (Agilent Technologies). In brief, mRNA was reverse transcribed and amplified using an oligo-dT-T7 promoter primer, and the resulting cRNA was labeled with cyanine 3–CTP or cyanine 5–CTP. After precipitation, purification, and quantification, 1.25 μg of each labeled cRNA was fragmented and hybridized to whole-genome mouse 4 × 44K multipack microarrays (Agilent-014868, Whole Mouse Genome 4 × 44K Microarray Kit) according to the supplier’s protocol (Agilent Technologies). Scanning of microarrays was performed at 5 μm resolution using a G2565CA high-resolution laser microarray scanner (Agilent Technologies) with an extended dynamic range (XDR). Microarray image data were analyzed with Image Analysis/Feature Extraction software G2567AA, version A.11.5.1.1 (Agilent Technologies), using default settings and the GE2_1105_Oct12 extraction protocol. The extracted MAGE-ML files were analyzed further with Rosetta Resolver Biosoftware, build 7.2.2 SP1.31 (Rosetta Biosoftware). Ratio profiles comprising single hybridizations were combined in an error-weighted fashion to create ratio experiments. A 1.5-fold change expression cut-off for ratio experiments was applied together with anticorrelation of dye-swapped ratio profiles, rendering the microarray analysis highly significant (*P* < 0.01), robust, and reproducible (48). Microarray data have been deposited in the NCBI’s Gene Expression Omnibus (GEO GSE145087).

### GSEA.

We performed GSEA on genes ranked by gene expression–based *t* score between gastric corpus epithelium isolated from *Rspo3*-KI and *Rspo3-*WT controls using the fgsea R package (49) with 5,000 permutations. We used a gene set of a stem cell signature obtained from LGR5^+^ cells in intestinal crypts (18), a gene set for genes associated with DNA biosynthesis (50), a gene set derived from gastric cancer samples of 32 patients (29), and a gene set for genes assigned to SPEM (28) as well as a gene set comparing the signature of regenerative Sca1^+^ epithelial cells during acute DSS-induced colitis to the signature of homeostatic epithelium (22). *P* values were adjusted for multiple testing by a global FDR according to the method described by Benjamini and Hochberg (51). The computational code for GSEA analysis of microarray in this manuscript data can be accessed under https://github.com/Sigal-Lab/Fischer_et_al_Corpus_Glands_Rspo3/tree/f644b8d (commit ID f644b8d).

### qPCR.

RNA was extracted from snap-frozen corpus tissue using the RNAeasy RNA Purification Kit (QIAGEN) including on-column DNase digestion. qPCR was performed using a Power SYBR Green RNA-to-CT 1-Step Kit (Applied Biosystems) according to the manufacturer’s instructions. Reactions were performed in a reaction mix of 25 μl containing 50 ng RNA, 12.5 μl SYBR Green mix, 0.16 μl RT mix, and 0.2 μM primer. Reactions were performed for 30 minutes at 48°C followed by 10 minutes at 95°C and then 40 cycles of 15 seconds at 95°C/60 seconds at 60°C.

For primers used in this study, see Table 1.

For each oligonucleotide pair and RNA sample, the reaction was performed in duplicate. The amplification plots obtained from RT-PCR were analyzed with the ABI Prism SDS Software package, version 2.2.2 (Applied Biosystems). The expression levels of the target genes were normalized to the levels of Gapdh expression in each sample.

### Quantification of cell numbers.

Quantification of cell numbers was performed semiautomatically. Immunofluorescence images were analyzed using QuPath software, version 0.2.3. The number of cells per image, as well as the number of GIF^+^ or GSII^+^ cells, was assessed. The number of GIF^+^GSII^+^ cells was assessed manually using the annotations obtained from the “positive cell analysis.”

### Inclusion/exclusion criteria.

Mice were excluded from the analysis if induction of *Rspo3* KO or *Rspo3* overexpression did not lead to a significant reduction or increase of *Rspo3* expression. For the analysis of corpus samples used for immunofluorescence, the data from all vertically cut glands from consecutive images taken from a longitudinal section through the corpus were used to exclude sampling errors.

### Data availability.

Microarray data from this manuscript have been deposited in the NCBI’s GEO database (GSE145087).

### Statistics.

Experiments were performed on a minimum of *n* = 3 biological replicates unless otherwise stated. For most mouse experiments, 5 to 10 mice per group were used. Microarray analysis was performed using samples from 2 biological replicates. All data are represented as mean ± SEM. Statistics are based on *n* biological replicates. For comparison of 2 groups, a 2-tailed, unpaired *t* test was performed. Two-tailed ordinary 1-way ANOVA with respective post hoc analysis (Tukey’s test) was used for statistical comparison of more than 2 groups. All analyses of statistical significance were calculated and displayed compared with the reference control group unless otherwise stated. *P* < 0.05 was considered significant. Statistical analysis was done using GraphPad Prism software, version 8.3.2.

### Study approval.

All procedures involving animals were approved by the institutional and local legal authorities at the Max Planck Institute for Infection Biology as well as at Charité-Universitätsmedizin Berlin (LAGeSo, Berlin). Paraffin sections from resected gastric cancer samples were received from the Charité-Universitätsmedizin Berlin Department of Pathology. Samples were deidentified, and retrospective analysis was authorized by local authorities. All human participants gave written, informed consent.

## Author contributions

The study was designed by ASF and MS. ASF performed most of the experiments and most of the data analysis. SM and ASF performed the animal procedures. ASF and SM performed in situ hybridization and qPCR gene expression analysis. JH provided critical intellectual support, HJM performed microarrays and analyzed the data, and HB analyzed data and performed gene enrichment analysis. JW and ML performed immunofluorescence stainings. AA and DH provided and analyzed human pathology samples, and FT provided reagents and infrastructure support. The manuscript was written by MS and ASF, and all authors read and edited the manuscript.

## Supplementary Material

Supplemental data

## Figures and Tables

**Table 1:**
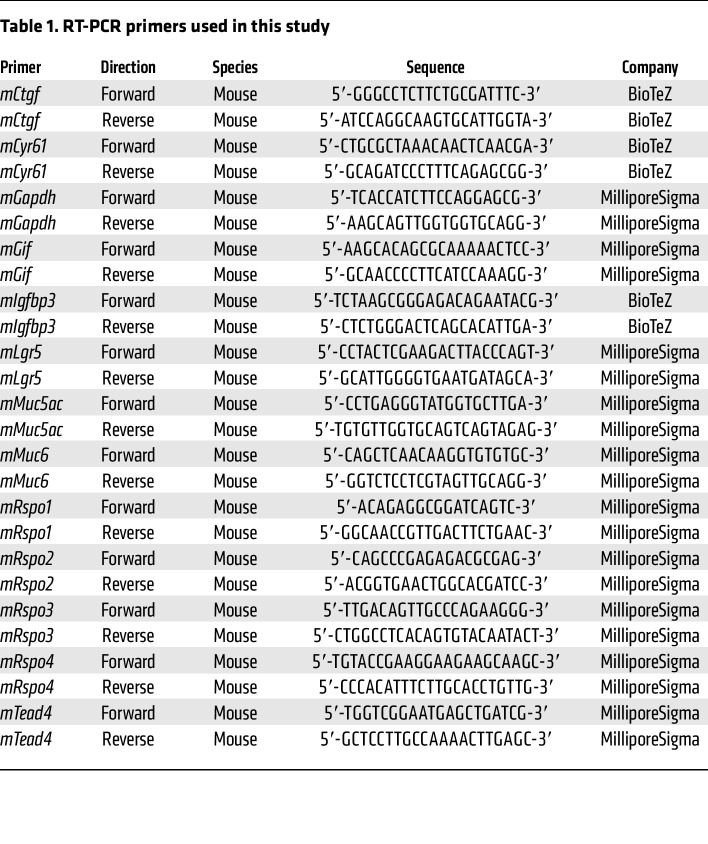
RT-PCR primers used in this study

## Version 1 09/13/2022

In-Press Preview

## Version 2 11/01/2022

Electronic publication

## References

[1] Demitrack ES (2017). NOTCH1 and NOTCH2 regulate epithelial cell proliferation in mouse and human gastric corpus. Am J Physiol Gastrointest Liver Physiol.

[2] Matsuo J (2017). Identification of stem cells in the epithelium of the stomach corpus and antrum of mice. Gastroenterology.

[3] Karam SM (2003). Defining epithelial cell progenitors in the human oxyntic mucosa. Stem Cells.

[4] Quante M (2010). TFF2 mRNA transcript expression marks a gland progenitor cell of the gastric oxyntic mucosa. Gastroenterology.

[5] Mills JC, Shivdasani RA (2011). Gastric epithelial stem cells. Gastroenterology.

[6] Wang TC (1998). Mice lacking secretory phospholipase A2 show altered apoptosis and differentiation with Helicobacter felis infection. Gastroenterology.

[7] Schmidt PH (1999). Identification of a metaplastic cell lineage associated with human gastric adenocarcinoma. Lab Invest.

[8] Yoshizawa N (2007). Emergence of spasmolytic polypeptide-expressing metaplasia in Mongolian gerbils infected with Helicobacter pylori. Lab Invest.

[9] Shimizu T (2016). Characterization of progressive metaplasia in the gastric corpus mucosa of Mongolian gerbils infected with Helicobacter pylori. J Pathol.

[10] Sigal M (2017). Stromal R-spondin orchestrates gastric epithelial stem cells and gland homeostasis. Nature.

[11] Sigal M (2019). R-spondin-3 induces secretory, antimicrobial LGR5^+^ cells in the stomach. Nat Cell Biol.

[12] Harnack C (2019). R-spondin 3 promotes stem cell recovery and epithelial regeneration in the colon. Nat Commun.

[13] Yan KS (2017). Non-equivalence of Wnt and R-spondin ligands during LGR5^+^ intestinal stem-cell self-renewal. Nature.

[14] Kim KA (2008). R-Spondin family members regulate the Wnt pathway by a common mechanism. Mol Biol Cell.

[15] Hilkens J (2017). RSPO3 expands intestinal stem cell and niche compartments and drives tumorigenesis. Gut.

[16] Leushacke M (2017). LGR5-expressing chief cells drive epithelial regeneration and cancer in the oxyntic stomach. Nat Cell Biol.

[17] Stange DE (2013). Differentiated Troy+ chief cells act as reserve stem cells to generate all lineages of the stomach epithelium. Cell.

[18] Munoz J (2012). The LGR5 intestinal stem cell signature: robust expression of proposed quiescent ‘+4’ cell markers. EMBO J.

[19] Kabiri Z (2014). Stroma provides an intestinal stem cell niche in the absence of epithelial Wnts. Development.

[20] Huh WJ (2012). Tamoxifen induces rapid, reversible atrophy, and metaplasia in mouse stomach. Gastroenterology.

[21] Keeley TM (2019). Tamoxifen-induced gastric injury: effects of dose and method of administration. Cell Mol Gastroenterol Hepatol.

[22] Yui S (2018). YAP/TAZ-dependent reprogramming of colonic epithelium links ECM remodeling to tissue regeneration. Cell Stem Cell.

[23] von Eyss B (2015). A MYC-driven change in mitochondrial dynamics limits YAP/TAZ function in mammary epithelial cells and breast cancer. Cancer Cell.

[24] Serra D (2019). Self-organization and symmetry breaking in intestinal organoid development. Nature.

[25] Parsonnet J (1991). Helicobacter pylori infection and the risk of gastric carcinoma. N Engl J Med.

[26] Huang JQ (2003). Meta-analysis of the relationship between cagA seropositivity and gastric cancer. Gastroenterology.

[27] [No authors listed] (1994). Schistosomes, liver flukes and Helicobacter pylori. IARC Working Group on the Evaluation of Carcinogenic Risks to Humans. Lyon, 7-14 June 1994. IARC Monogr Eval Carcinog Risks Hum.

[28] Nozaki K (2008). A molecular signature of gastric metaplasia arising in response to acute parietal cell loss. Gastroenterology.

[29] Vecchi M (2007). Gene expression analysis of early and advanced gastric cancers. Oncogene.

[30] Greicius G (2018). *PDGFRα*^+^ pericryptal stromal cells are the critical source of Wnts and RSPO3 for murine intestinal stem cells in vivo. Proc Natl Acad Sci U S A.

[31] Hata M (2020). GPR30-expressing gastric chief cells do not dedifferentiate but are eliminated via PDK-dependent cell competition during development of metaplasia. Gastroenterology.

[32] Caldwell B (2021). Chief cell plasticity is the origin of metaplasia following acute injury in the stomach mucosa. Gut.

[33] Fischer AS, Sigal M (2019). The role of Wnt and R-spondin in the stomach during health and disease. Biomedicines.

[34] Neumeyer V (2019). Mutated *Rnf43* aggravates *Helicobacter Pylori*-induced gastric pathology. Cancers (Basel).

[35] Kapalczynska M (2022). BMP feed-forward loop promotes terminal differentiation in gastric glands and is interrupted by *H*. *pylori*-driven inflammation. Nat Commun.

[36] Gregorieff A (2015). Yap-dependent reprogramming of LGR5(+) stem cells drives intestinal regeneration and cancer. Nature.

[37] Dupont S (2011). Role of YAP/TAZ in mechanotransduction. Nature.

[38] Aragona M (2013). A mechanical checkpoint controls multicellular growth through YAP/TAZ regulation by actin-processing factors. Cell.

[39] Khurana SS (2013). The hyaluronic acid receptor CD44 coordinates normal and metaplastic gastric epithelial progenitor cell proliferation. J Biol Chem.

[40] Willet SG (2018). Regenerative proliferation of differentiated cells by mTORC1-dependent paligenosis. EMBO J.

[41] Petersen CP (2018). A signaling cascade of IL-33 to IL-13 regulates metaplasia in the mouse stomach. Gut.

[42] Meyer AR (2019). Cystine/glutamate antiporter (xCT) is required for chief cell plasticity after gastric injury. Cell Mol Gastroenterol Hepatol.

[43] Barker N (2007). Identification of stem cells in small intestine and colon by marker gene LGR5. Nature.

[44] Tian H (2011). A reserve stem cell population in small intestine renders LGR5-positive cells dispensable. Nature.

[45] Neufeld S (2012). A conditional allele of Rspo3 reveals redundant function of R-spondins during mouse limb development. Genesis.

[46] Herring BP (2014). Previously differentiated medial vascular smooth muscle cells contribute to neointima formation following vascular injury. Vasc Cell.

[47] Arnold IC (2011). Tolerance rather than immunity protects from Helicobacter pylori-induced gastric preneoplasia. Gastroenterology.

[48] Churchill GA (2002). Fundamentals of experimental design for cDNA microarrays. Nat Genet.

[50] Liberzon A (2011). Molecular signatures database (MSigDB) 3.0. Bioinformatics.

[51] Benjamini Y, Hochberg Y (1995). Controlling the false discovery rate: a practical and powerful approach to multiple testing. J R Stat Soc.

