# Supplementary material for: R-spondin/YAP axis promotes gastric oxyntic gland regeneration and Helicobacter pylori–associated metaplasia in mice
Source: J Clin Invest. 2022 Nov 1;132(21):e151363. doi: 10.1172/JCI151363 (PMC9621134; doi:10.1172/JCI151363)
Supplement: Supplemental data [file jci-132-151363-s147.pdf]

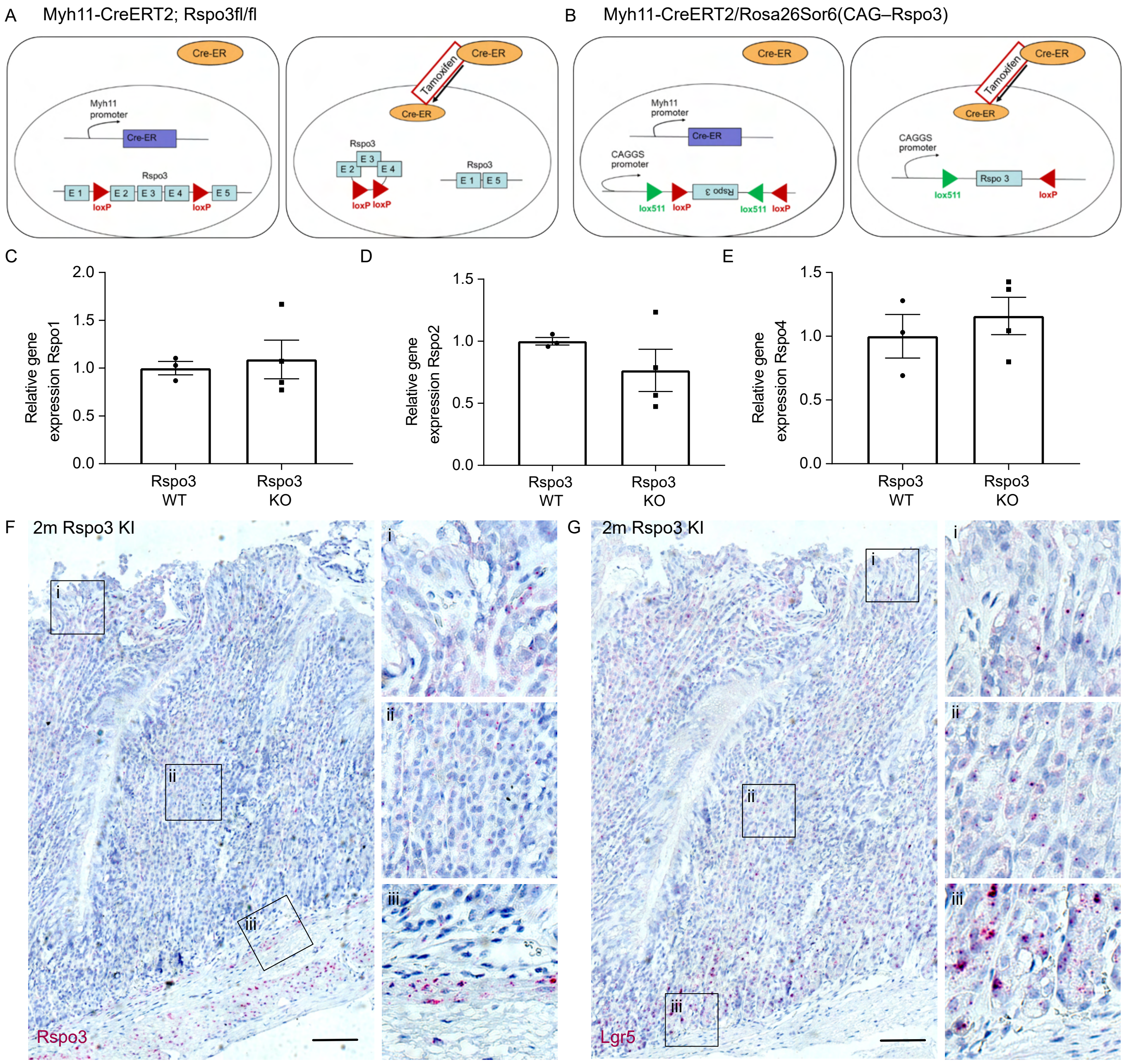

Supplementary Figure 1: Conditional mouse models enable adjustment of myofibroblast-derived *Rspo3* expression while maintaining the expression pattern of *R-spondin* isoforms and *Lgr5* gradient

(A) Scheme of *Myh11-CreERT2; Rspo3*$^{fl/fl}$ mice (adapted from Neufeld et al(45)). (B) *Myh11-CreERT2/Rosa26Sor6(CAG–Rspo3)* (adapted from Hilkens et al(15)). (C-E) qPCR data showing expression of (C) *Rspo1*, (D) *Rspo2*, and (E) *Rspo4* in the corpus of *Rspo3* KO (n=4 mice) versus WT mice (n=3 mice). (F) ISH of *Rspo3* (red) in the corpus tissue of a *Rspo3* KI mouse. (G) ISH of *Lgr5* (red) in the corpus tissue of a *Rspo3* KI mouse. Scale bar: 100 µm. Unpaired parametric t-test.

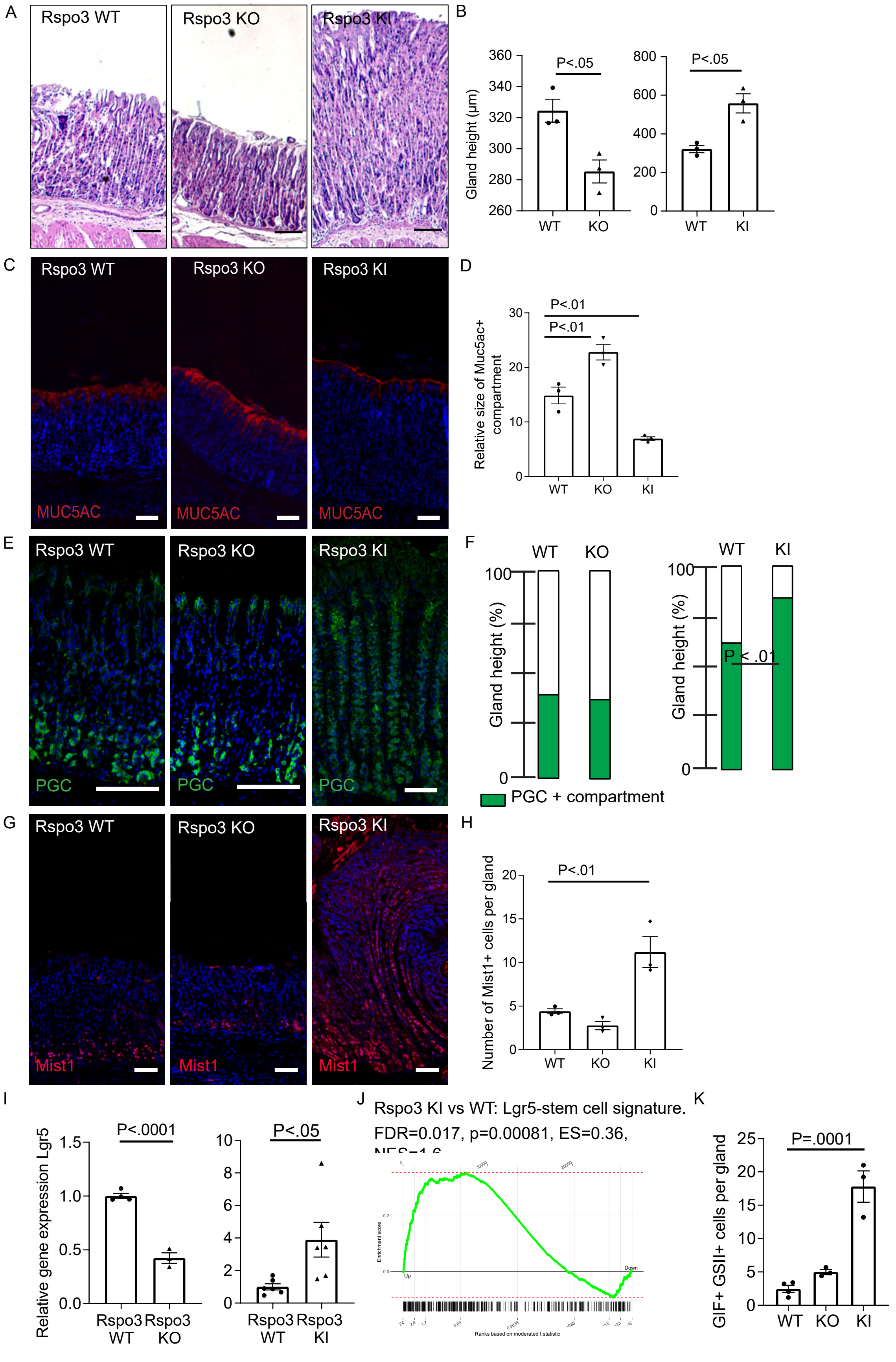

Supplementary Figure 2: Effect of RSPO3 on epithelial cell populations in the corpus

(A) H&E staining representative of Rspo3 WT mice, Rspo3 KO mice, and Rspo3 KI mice. (B) Gland height in µm in Rspo3 KO and Rspo3 KI mice and littermate controls (n=3 mice per group). (C) Immunofluorescence images of MUC5AC labeling (red) representative of Rspo3 KO and Rspo3 KI mice and corresponding littermate controls. (D) Relative size of the MUC5AC+ gland compartment in Rspo3 KO and Rspo3 KI mice and corresponding littermate controls (n=3 mice per group). (E) Immunofluorescence images of pepsinogen C (PGC) (green) labeling in sections representative of Rspo3 KO and Rspo3 KI mice and corresponding littermate controls. The image shown for Rspo3 WT was taken from the same section as the image shown in Figure 1D. (F) Bar chart visualizing the location and relative size of the PGC positive gland compartment normalized to the gland height and expressed as % of Rspo3 KO and Rspo3 KI mice and corresponding littermate controls (n=3 mice per group). (G) Immunofluorescence images of Mist1 (red) labeling in sections representative of Rspo3 KO and Rspo3 KI mice and corresponding littermate controls. (H) Number of Mist1 positive cells per gland (n=3 mice per group). (I) qPCR for Lgr5 expression of Rspo3 KO (n=6 mice) and Rspo3 KI mice (n=6 mice) versus littermate controls (n=6 mice per group). (J) GSEA of microarray data comparing the expression profile of corpus tissue from Rspo3 KI mice and littermate controls with a published intestinal Lgr5 signature (18). ES, enrichment score; NES, normalized enrichment score. (K) Quantification of GIF+GSII+ cells per gland in Rspo3 KO and Rspo3 KI mice and corresponding littermate controls (n=3 mice per group). Scale bars: 100 µm. Unpaired parametric t-test (B, F, H, I), one-way ANOVA with Tukey`s multiple comparison test (D, K).

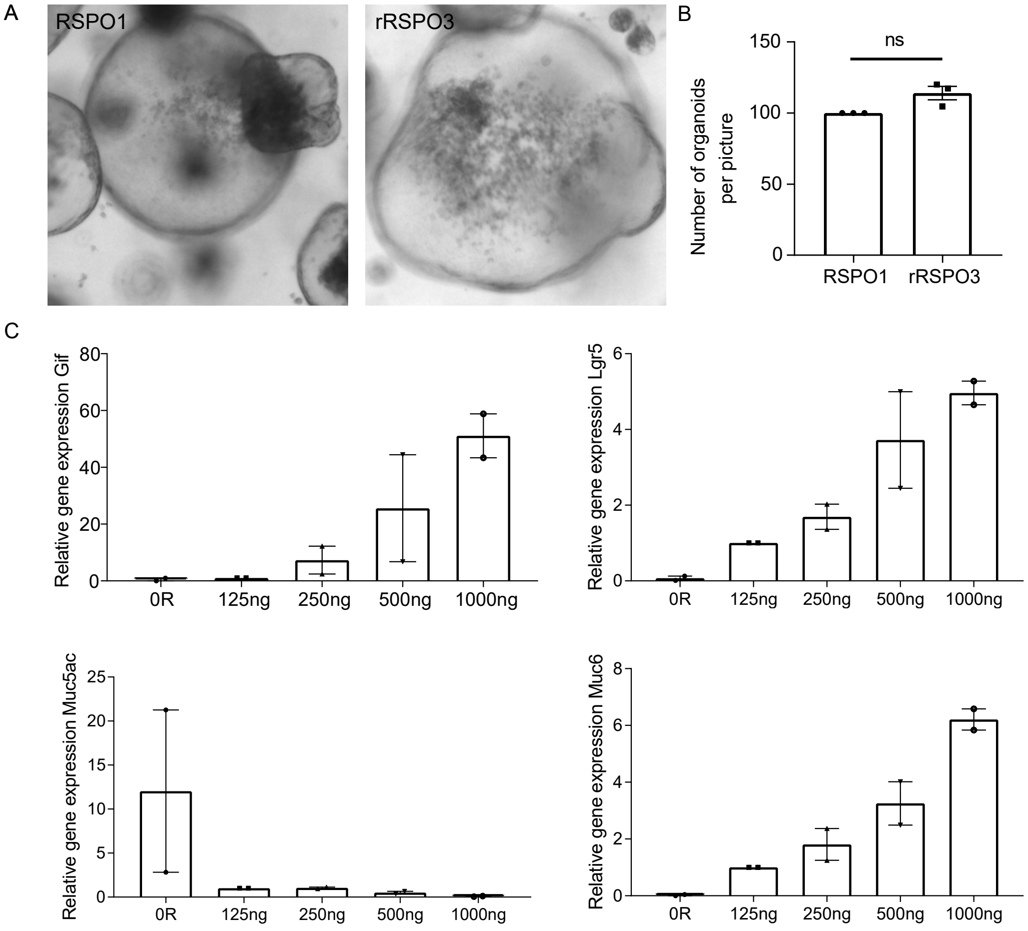

Supplementary Figure 3: Organoids grown in RSPO1 conditioned medium resemble organoids grown with recombinant RSPO3

(A) Representative images of organoids grown in RSPO1 conditioned medium and organoids grown with recombinant RSPO3 (rRSPO3). (B) Quantification of the number of organoids per image of cultures grown in RSPO1 conditioned medium versus cultures grown with recombinant Rspo3 (rRSPO3). n=3 replicates per group. (C) qPCR data showing expression of *Gif*, *Lgr5*, *Muc5ac,* and *Muc6* in organoids grown from murine corpus tissue cultured with different concentrations of recombinant RSPO3 (in ng/ml medium) (n=2 replicates). Unpaired parametric t-test (B).

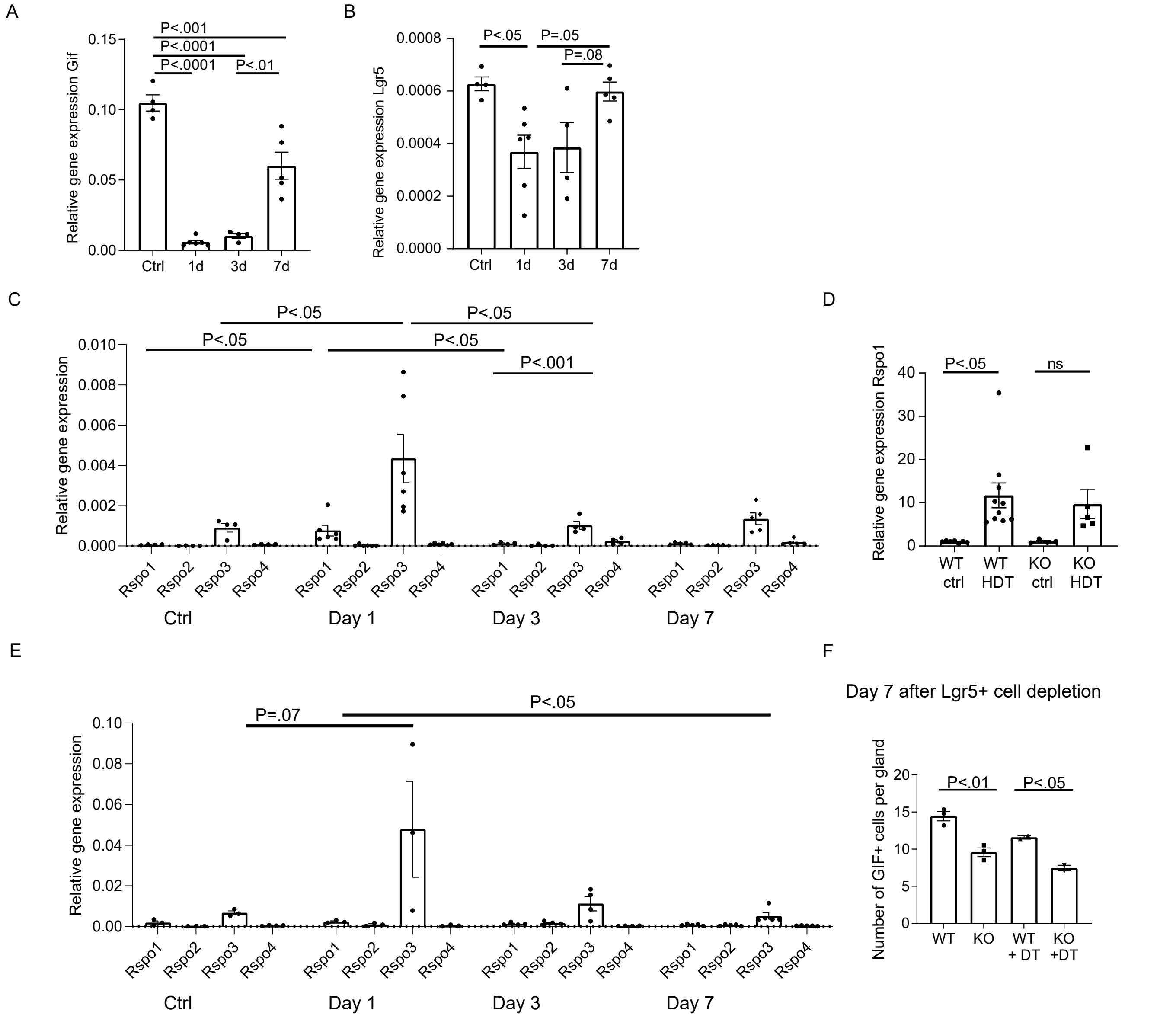

Supplementary Figure 4: Upon chief cell loss, *Rspo3* expression is upregulated
(A-C) qPCR data showing expression of (A) *Gif*, (B) *Lgr5*, and (C) *Rspo1-4* in the corpus of *Rspo3* WT mice treated with high-dose tamoxifen for 2 days and sacrificed on day 1 (n=6 mice), 3 (n=4 mice) or 7 (n=4 mice) versus non-treated controls (n=4 mice). C includes data shown in Figure 3C. (D) qPCR data showing expression of *Rspo1* in the corpus of *Rspo3* KO (n=5 mice) and *Rspo3* WT mice (n=10 mice) treated with high-dose tamoxifen for 2 days and sacrificed on day 1 and non-treated controls (n=4 *Rspo3* KO mice; n=7 *Rspo3* WT mice). Graph includes data shown in Figure 3P. (E) qPCR data showing expression of *Rspo1-4* in the corpus of Lgr5DTR mice treated with DT for 3 days and sacrificed on day 1 (n=3 mice), 3 (n=4 mice), or 7 (n=5 mice) versus controls (n=3 mice). (F) Quantification of the number of GIF+ cells per gland on day 7 after DT-induced depletion of Lgr5+ cells in *Lgr5DTR; Rspo3* WT and *Lgr5DTR; Rspo3* KO mice and non-treated controls. One-way ANOVA with Tukey`s multiple comparison test.

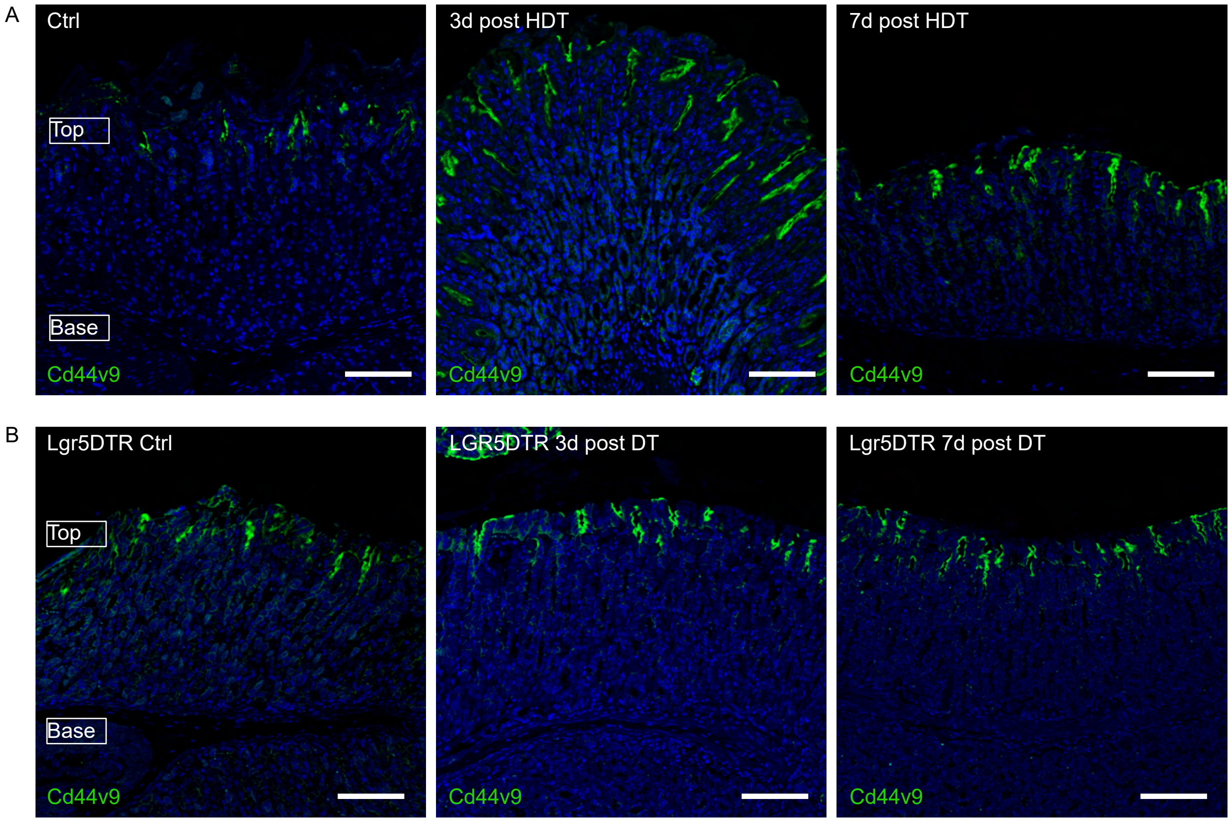

Supplementary Figure 5: Recovery after acute injury is driven by transitional GIF+ GSII+ cells instead of Cd44v9+ SPEM cells.
(A) Immunofluorescence staining for Cd44v9 (green) on tissue sections representative of Rspo3 WT mice treated with HDT for 2 days and sacrificed on day 3 or 7 versus controls. (B) Immunofluorescence staining for Cd44v9 (green) on tissue sections representative of Lgr5DTR mice treated with DT for 3 days and sacrificed on day 3 or 7 versus controls. Note: Signal in the pit cells is unspecific signal.

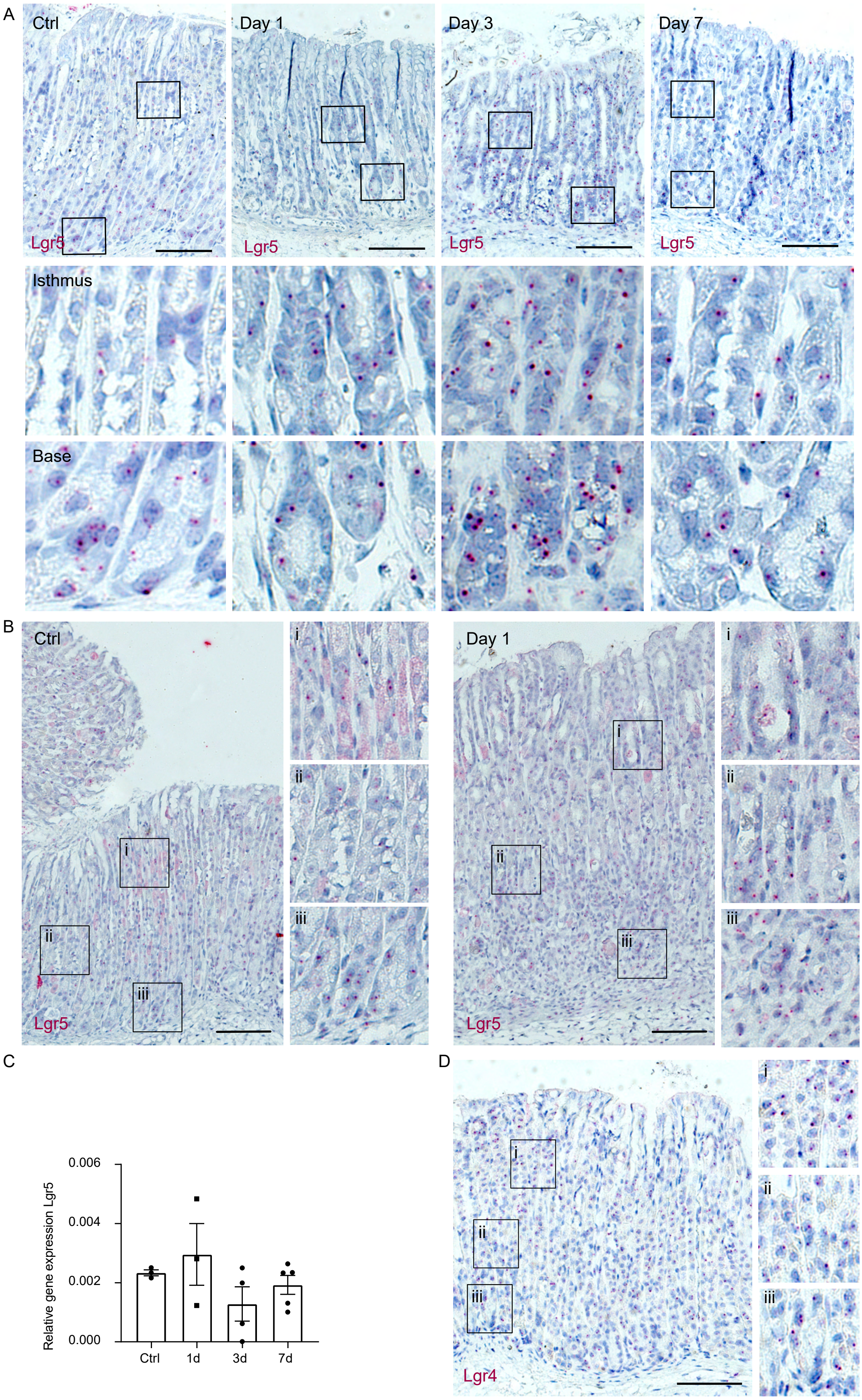

Supplementary Figure 6: Upon chief cell loss *Lgr5* expression in the gland and the isthmus is markedly increased
(A) ISH for *Lgr5* (red) on tissue sections from mice treated with high-dose tamoxifen on two consecutive days and sacrificed 1, 3, or 7 days after injury and non-treated control mice. (B) ISH for *Lgr5* (red) on tissue sections representative of Lgr5DTR mice treated with DT for 3 days and sacrificed on day 1 versus controls. (C) qPCR data showing expression of *Lgr5* in the corpus of Lgr5DTR mice treated with DT for 3 days and sacrificed on day 1 (n=3 mice), 3 (n=4 mice), or 7 (n=5 mice) versus controls (n=3 mice). (D) ISH for *Lgr4* (red) representative of non-treated Bl6 mice. Scale bars: 50 µm. One-way ANOVA with Tukey`s multiple comparison test.

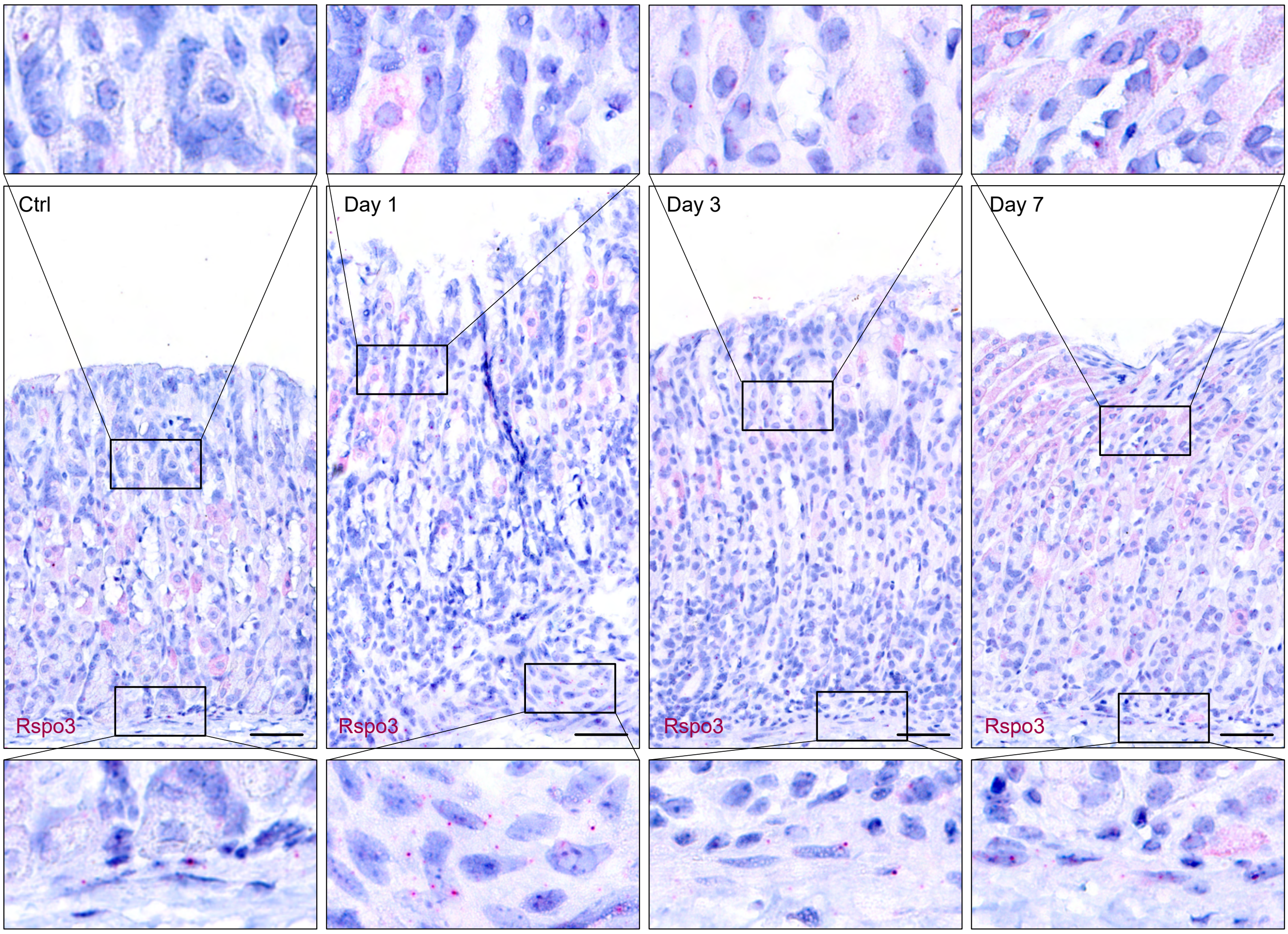

Supplementary Figure 7: Upon loss of chief cells, expression of *Rspo3* is predominantly upregulated in the stroma beneath the gland base with low expression beneath the isthmus

ISH for *Rspo3* (red) on tissue sections representative of Lgr5DTR mice treated with DT for 3 days and sacrificed on days 1, 3, and 7 versus controls.

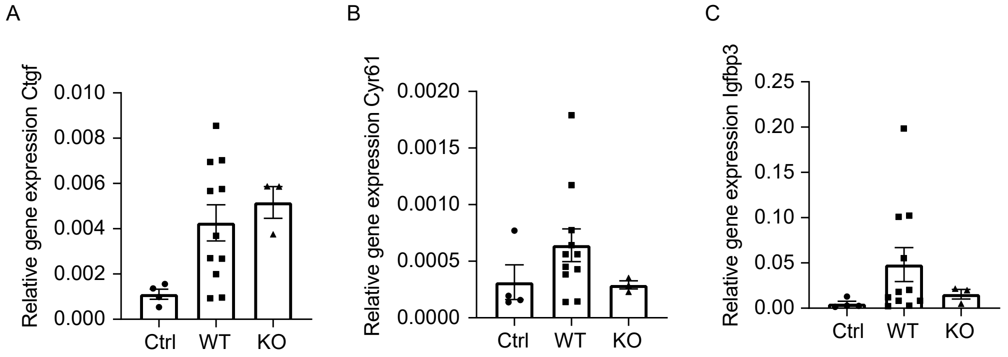

Supplementary Figure 8: Upon injury, YAP target gene expression shows reduced upregulation in *Rspo3* KO mice compared to *Rspo3* WT mice while upregulation of *Rspo1* expression is similar between WT and KO

(A-C) qPCR data showing expression of (A) *Ctgf*, (B) *Cyr61*, and (C) *Igfbp3* in the corpus of *Rspo3* WT mice treated with high-dose tamoxifen for 2 days and sacrificed on day 1 (n=6 mice), versus high-dose tamoxifen-treated *Rspo3* KO mice (n=5) and non-treated controls (n=4 mice).

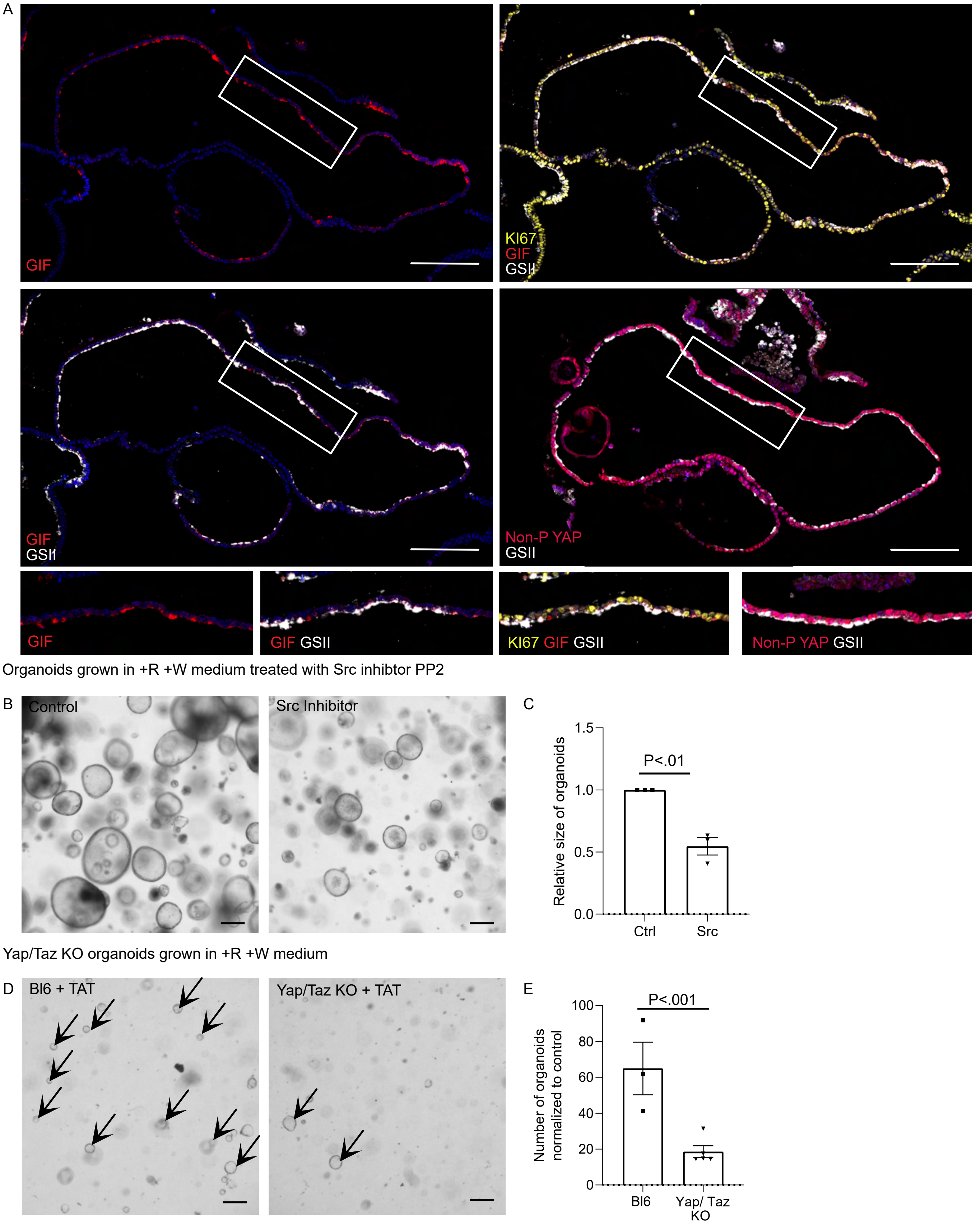

Supplementary Figure 9: Inhibition of YAP signaling leads to reduced proliferative capacity of organoids
All organoids were grown in 10% RSPO1 conditioned medium.
(A) Representative immunofluorescence images of GIF (red), GSII (grey), KI67 (yellow), and non-phospho YAP (pink) on sections of organoids grown in full medium. (B) Images of organoids grown in full medium treated with the Src inhibitor PP2 for two days and non-treated control organoids. (C) Quantification of the relative diameter of organoids grown as in (B) (n=3 replicates per group). (D) Images of Yap/Taz $^{fl/fl}$ organoids treated with TAT-Cre recombinase to induce the p-lox system versus control Bl6 organoids treated with TAT to examine toxic side effects of the treatment. Images show day 2 after passaging (and TAT treatment). (E) Quantification of the number of organoids grown per image from organoid cultures of TAT-Cre recombinase-induced Yap/Taz $^{fl/fl}$ organoids (n=3 replicates) and TAT-treated Bl6 organoids (n=4 replicates). Numbers display the percentage of organoid numbers normalized to corresponding non-treated Yap/Taz $^{fl/fl}$ or Bl6 organoid cultures. Unpaired parametric t-test.

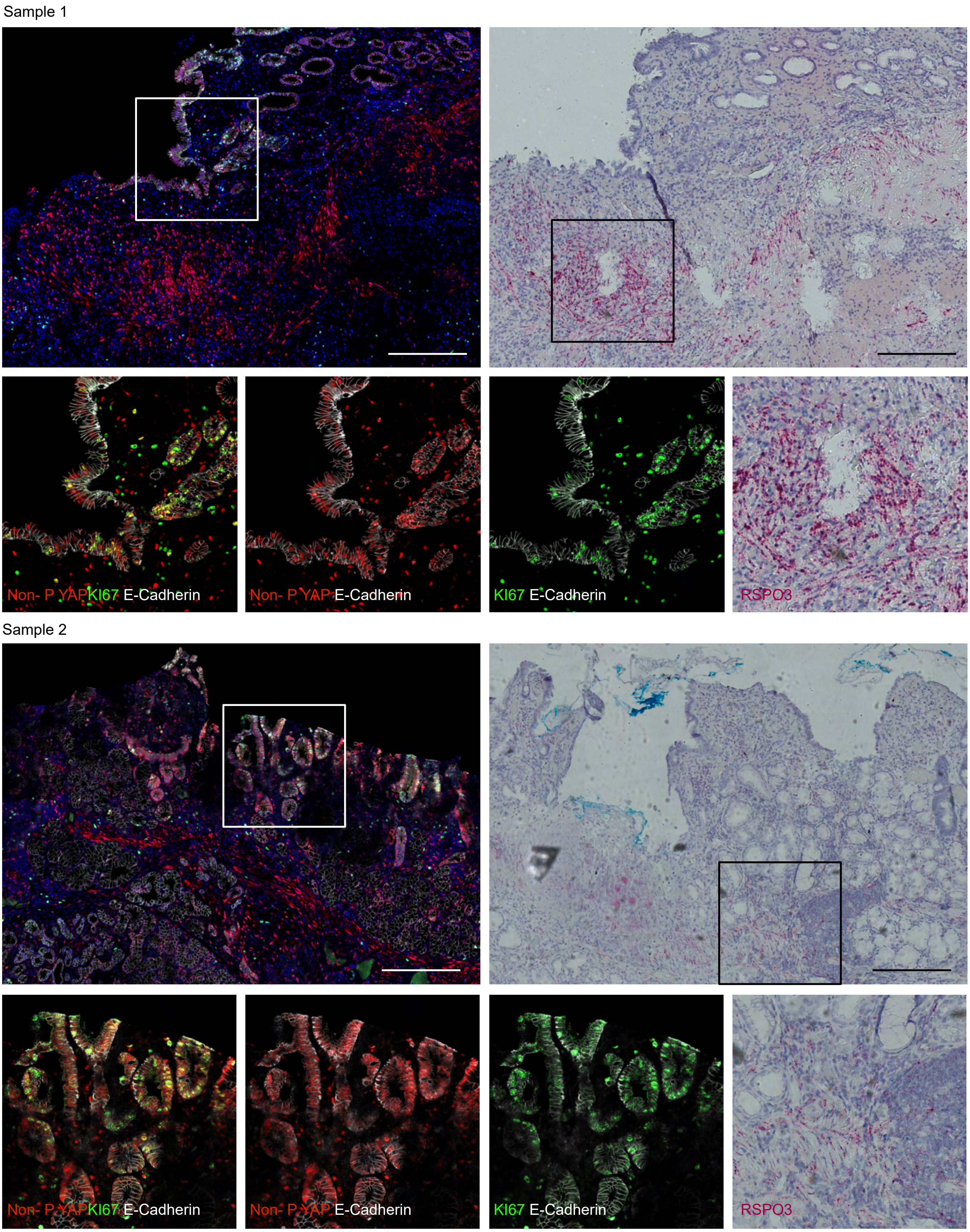

Supplementary Figure 10: In human gastric ulcer samples, stromal cells at the ulcer margin show high expression of *RSPO3,* and adjacent epithelial cells show nuclear (=active) YAP protein

Immunofluorescence staining for non-phospho YAP (red), KI67 (green) and E-Cadherin (grey), and ISH for RSPO3 (red) on sections of two human ulcer samples.

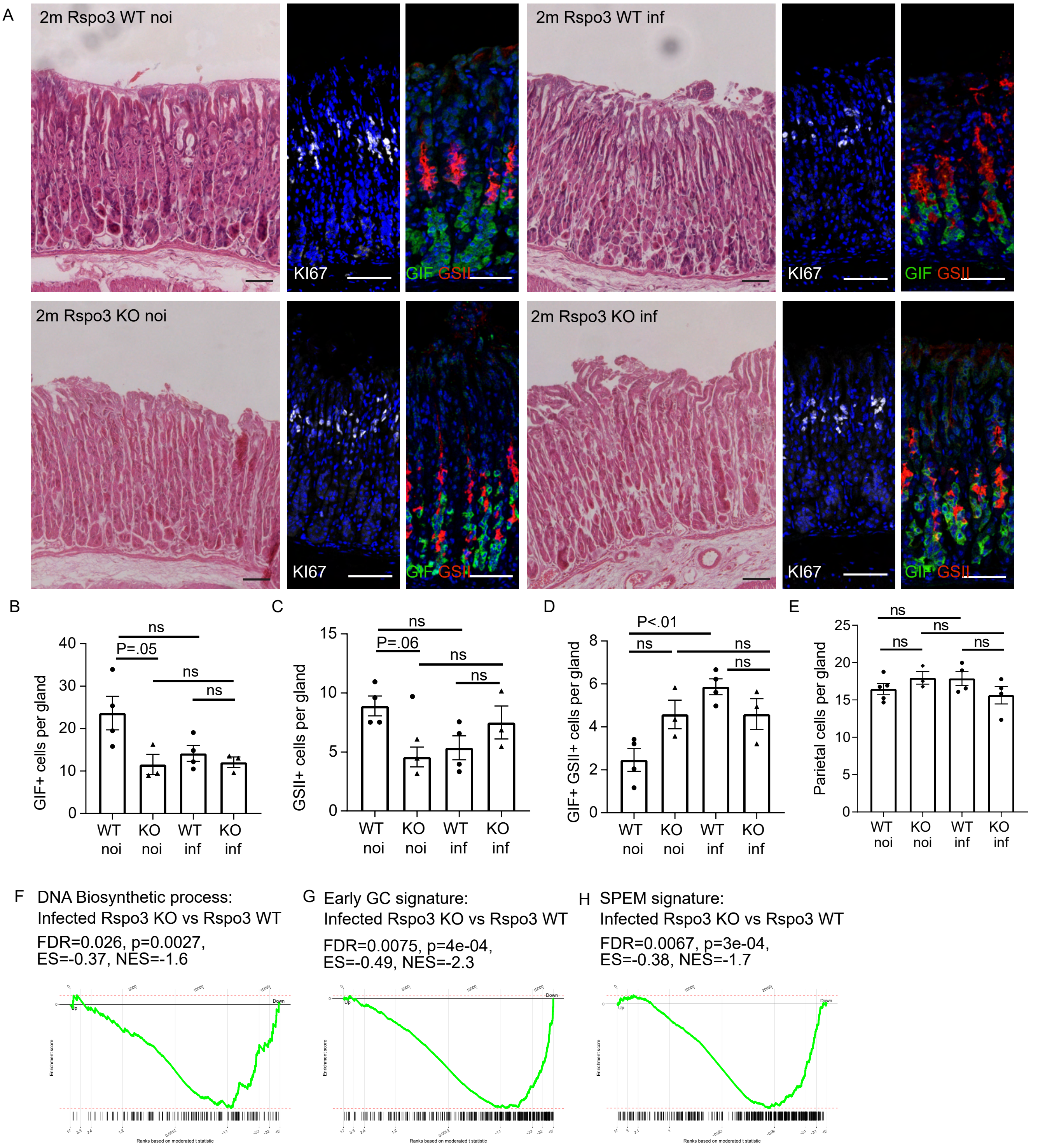

Supplementary Figure 11: Regenerative phenotype seen in infected *Rspo3* knock-in mice does not occur in infected *Rspo3* wild-type or *Rspo3* knockout mice (A) H&E staining and immunofluorescence staining for KI67 (white), GIF (green), and GSII (red) from 2-month infected *Rspo3* WT and *Rspo3* KO mice and non-infected littermate controls. Image shown for Rspo3 WT non-infected was taken from the same section as an image in Figure 1D. Scale bars: 50 µm. (B-E) Quantification of the number of (B) GIF+, (C) GSII+, (D) GIF+GSII+, and (E) parietal cells per gland in infected *Rspo3* KO and WT mice and non-infected controls (n=3 mice per group). Data for the control group equals data shown in Figure 1I, L, and Supplementary Figure 2K respectively. (F-H) GSEA of microarray data comparing the expression profile of the corpus tissue from 2-month infected *Rspo3* KO mice and infected littermate controls treated with tamoxifen 2 months before euthanasia with published data sets for (F) DNA biosynthetic process (50). (G) Early gastric cancer signature (29). (H) SPEM signature (28). ES, enrichment score; NES, normalized enrichment score. For GSEA n=2 mice per group. One-way ANOVA with Tukey`s multiple comparison test (A, I, L).

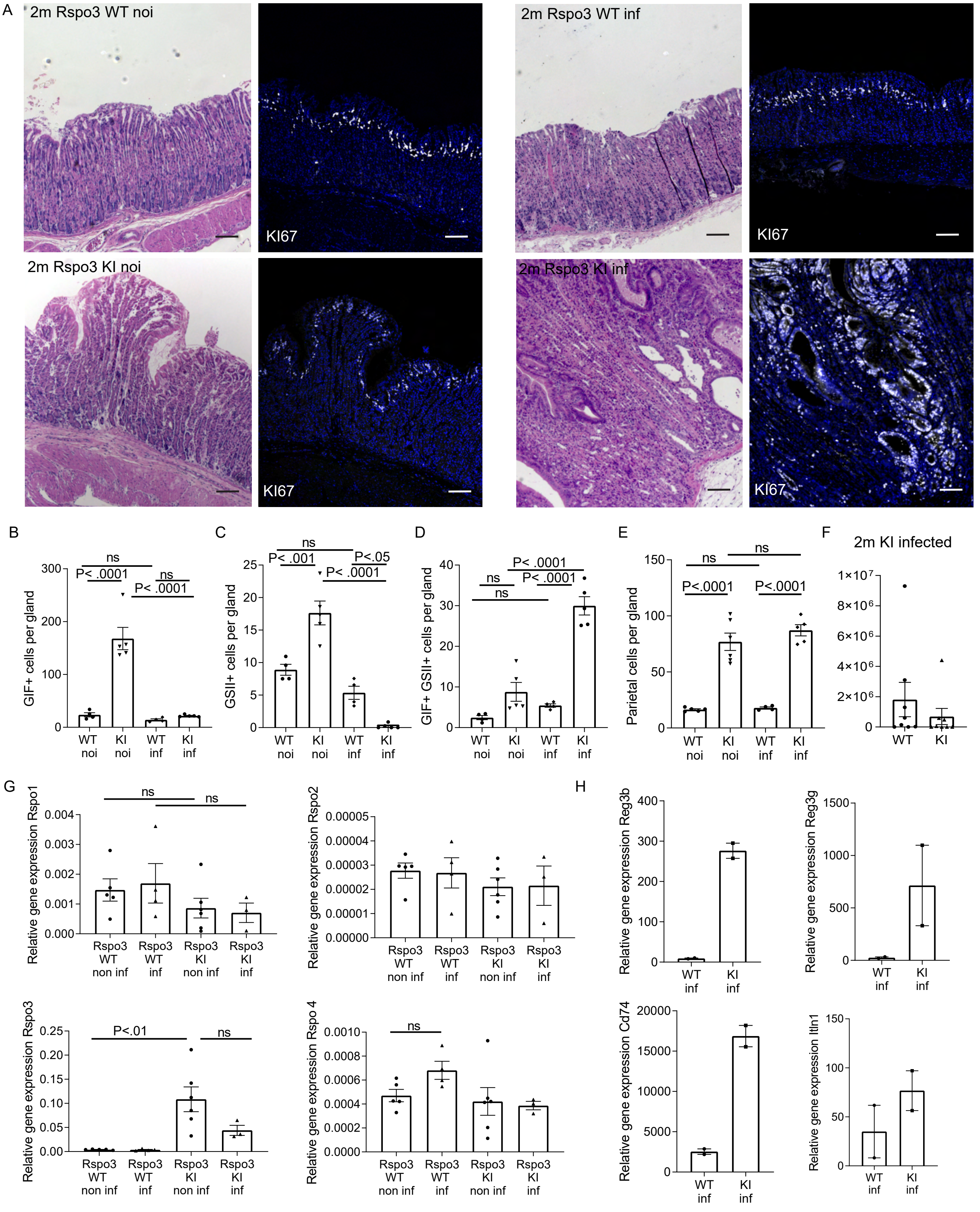

Supplementary Figure 12: Regenerative phenotype seen in infected *Rspo3* knock-in mice does not occur in non-infected knock-in mice or infected WT mice (A) H&E staining and immunofluorescence staining for KI67 (white) from 2-month infected *Rspo3* WT and *Rspo3* KI mice and non-infected littermate controls. Image shown for *Rspo3* KI inf is a different crop from Figure 6A. (B-E) Quantification of the number of (B) GIF+, (C) GSII+, (D) GIF+GSII+, and (E) parietal cells per gland in infected *Rspo3* KI and WT mice and non-infected controls (n=3 mice per group). Data for the control group equals data shown in Figure 1I, L, and Supplementary Figure 2K, 10 B-D, respectively. (F) Number of colony-forming units per gram stomach in 2-month infected *Rspo3* KI and WT mice. (G) qPCR gene expression data visualizing the expression of *Rspo1, 2, 3,* and *4* in the corpus of infected *Rspo3* KI and WT mice and non-infected controls. (H) Microarray gene expression data visualizing the expression level of *Reg3b*, *Reg3g*, *Cd7,4,* and *Itln1* in the corpus of infected *Rspo3* KI vs infected *Rspo3* WT mice. Scale bars: 100 µm. Unpaired parametric t-test (F), one-way ANOVA with Tukey`s multiple comparison test (B-E, G).

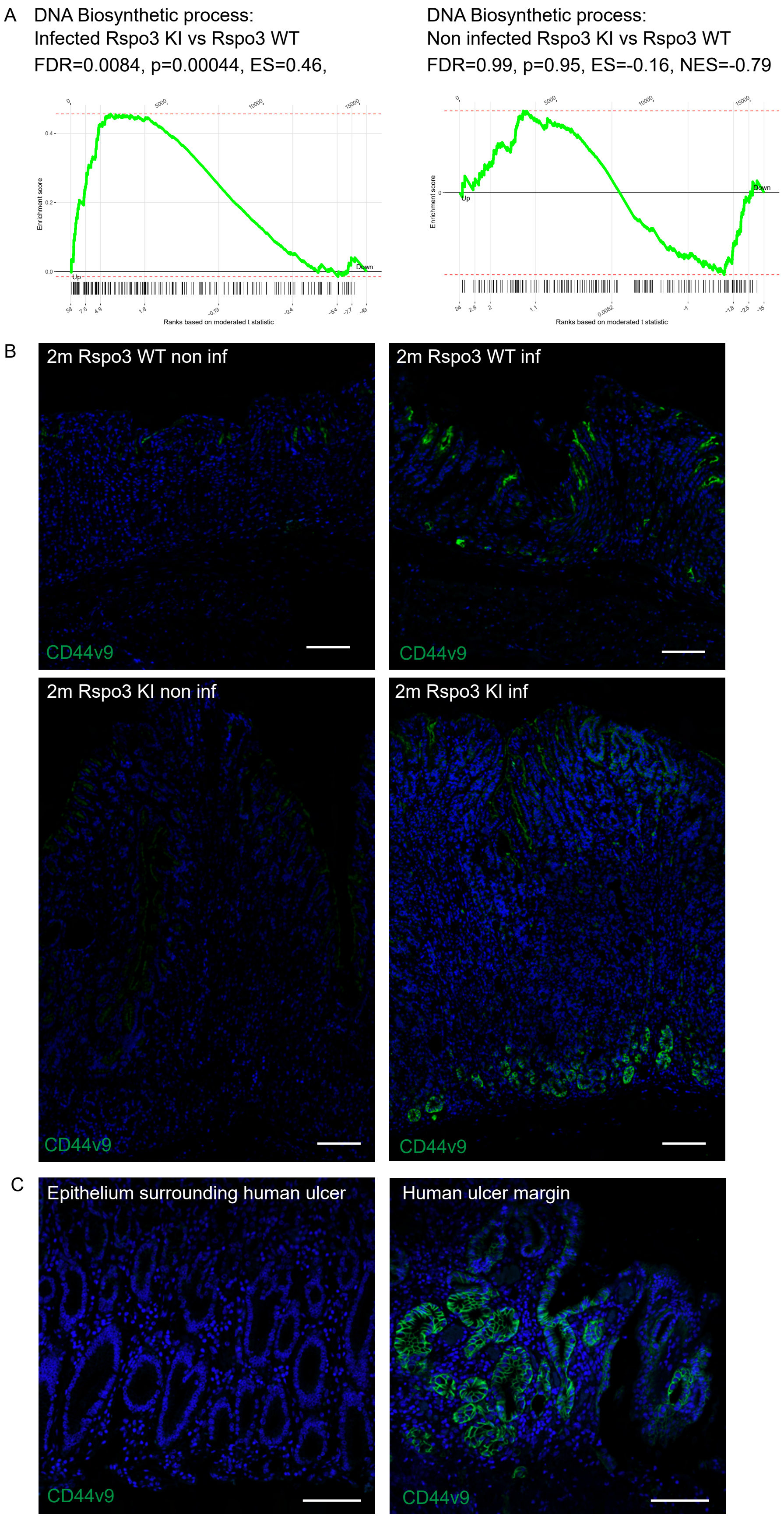

Supplementary Figure 13: Infected Rspo3 overexpressing mice show gene expression patterns assigned to DNA synthesis and SPEM
(A) GSEA of microarray data comparing the expression profile of the corpus tissue from 2-month infected Rspo3 KI mice and infected littermate controls as well as of non-infected Rspo3 KI mice and littermate controls with a published data set for DNA biosynthetic process. n=2 mice per group. (B) Immunofluorescence images of CD44v9 (green) of sections representative of 2-month infected Rspo3 KI mice and Rspo3 WT mice and non-infected littermate controls. (C) Immunofluorescence images of CD44v9 (green) of human ulcer samples.
Scale bar: 100 µm.

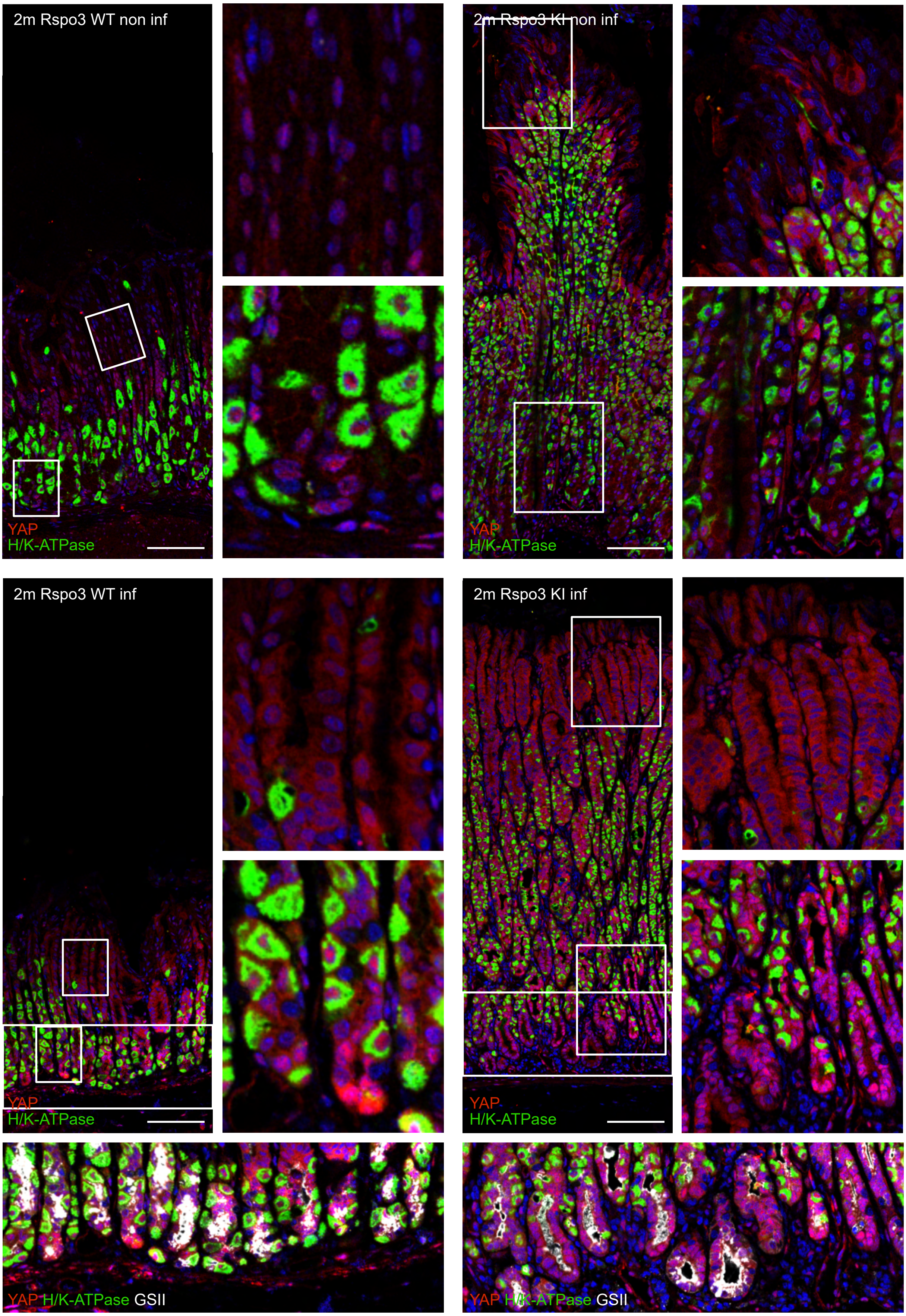

Supplementary Figure 14: YAP protein is expressed in GSII+ gland base cells in infected *Rspo3* overexpressing mice
Immunofluorescence images of YAP (red), GSII (grey), and H/K-ATPase (green) of a section representative of 2-month infected *Rspo3* KI mice. Scale bar: 25 µm.

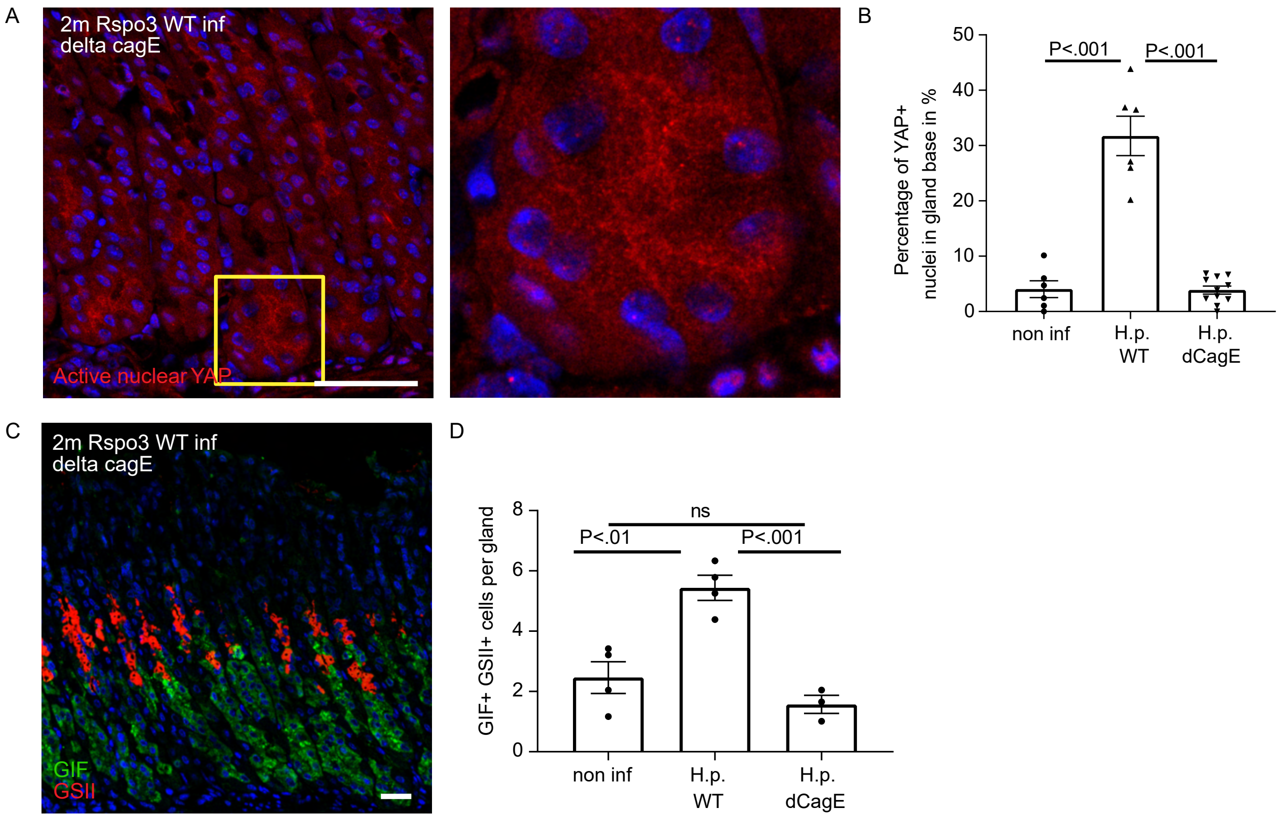

Supplementary Figure 15: Nuclear expression of YAP upon *H. pylori* infection is dependent on an intact type IV secretion system
(A) Representative immunofluorescence images of non-phospho (=active nuclear) YAP (red) of sections from *Rspo3* WT mice infected for 2 months with a type IV secretion system-deficient *H. pylori* strain (dCagE). (B)Percentage of YAP+ nuclei in gland bases of sections from *Rspo3* WT mice infected with a type IV secretion system deficient *H. pylori* strain (dCagE) compared to non-infected WT mice and *H. pylori* strain PMSS1 (intact type IV secretion system) infected mice. Data for control groups equal data from Fig. 7F. (C) Immunofluorescence image of GSII (red) and GIF (green) representative of sections from *Rspo3* WT mice infected for 2 months with a type IV secretion system-deficient *H. pylori* strain (dCagE). (D) Quantification of the number of GIF+GSII+ cells per gland of *Rspo3* WT mice infected for 2 months with the *H. pylori* PMSS1 strain (n=4 mice), *Rspo3* WT mice infected with a type IV secretion system-deficient *H. pylori* strain (dCagE) for 2 months (n=3 mice) and non-infected *Rspo3* WT control mice (n=4 mice). Scale bar: 50µm. One-way ANOVA with Tukey`s multiple comparison test (A, I, L).
